# Transplantation of enteric nervous system stem cells rescues nitric oxide synthase deficient mouse colon

**DOI:** 10.1038/ncomms15937

**Published:** 2017-07-03

**Authors:** Conor J. McCann, Julie E. Cooper, Dipa Natarajan, Benjamin Jevans, Laura E. Burnett, Alan J. Burns, Nikhil Thapar

**Affiliations:** 1 Stem Cells and Regenerative Medicine, UCL Great Ormond Street Institute of Child Health, 30 Guilford Street, London WC1N, UK; 2 Department of Clinical Genetics, Erasmus Medical Center, Rotterdam 3015 CN, The Netherlands

## Abstract

Enteric nervous system neuropathy causes a wide range of severe gut motility disorders. Cell replacement of lost neurons using enteric neural stem cells (ENSC) is a possible therapy for these life-limiting disorders. Here we show rescue of gut motility after ENSC transplantation in a mouse model of human enteric neuropathy, the neuronal nitric oxide synthase (*nNOS*^*−/−*^) deficient mouse model, which displays slow transit in the colon. We further show that transplantation of ENSC into the colon rescues impaired colonic motility with formation of extensive networks of transplanted cells, including the development of nNOS^+^ neurons and subsequent restoration of nitrergic responses. Moreover, post-transplantation non-cell-autonomous mechanisms restore the numbers of interstitial cells of Cajal that are reduced in the *nNOS*^*−/−*^ colon. These results provide the first direct evidence that ENSC transplantation can modulate the enteric neuromuscular syncytium to restore function, at the organ level, in a dysmotile gastrointestinal disease model.

## Abstract (web summary)

Isolated human and mouse enteric nervous system stem cells (ENSCs) are capable of integrating and promoting innervation of the mouse colon. Here the authors show that transplantation of mouse ENSCs into a mouse model of human enteric neuropathy restores colon motility.

Neuropathological loss within the enteric nervous system (ENS) has been implicated in a wide range of severe gut motility disorders, such as achalasia^1^,^2^,^3^, gastroparesis^4^,^5^, slow transit constipation^6^,^7^,^8^ and Hirschsprung’s disease (aganglionic megacolon)^9^,^10^, as well as being associated with a number of central nervous system disorders^11^,^12^,^13^. Potential replacement of lost neurons using stem cell replacement is an attractive therapy for such life-limiting disorders. Enteric neural stem cells (ENSC), which exist in both embryonic and adult gut, have been suggested as potential cell source for such treatments^14^,^15^. We and others have previously demonstrated the potential of both mouse^16^,^17^ and human^18^ ENSC to integrate within wild-type ganglionated mouse colon. Yet a limiting factor in the advancement of ENSC therapies for human application has been the failure to demonstrate functional rescue of motility in pathological disease models. Recent studies have demonstrated the successful integration of murine and human ENSC within aganglionic colon both *in vivo*^16^,^19^ and *ex vivo*^20^,^21^; however, the severity of the gut phenotype and poor survival of homozygote mice has limited their *in vivo* use for investigating the potential functional rescue, at the organ level, of ENSC-based therapies.

Other models of neuronal loss are, therefore, essential to test the viability of cell-based transplantation techniques to restore functional deficits resulting from neuropathology. The loss of neuronal nitric oxide synthase (nNOS) has been implicated in a range of human enteric neuropathies^22^, including oesophageal achalasia^23^, infantile hypertrophic pyloric stenosis^24^, gastroparesis (idiopathic and diabetic)^25^, colonic dysfunction^26^ and Hirschsprung’s disease^27^,^28^. Notably, *nNOS*^*−/−*^ mice recapitulate the clinical phenotype of a number of human diseases displaying both delayed gastric emptying^29^,^30^,^31^, and slow transit in the colon^32^ hence providing an ideal model to establish if ENSC can restore function after *in vivo* transplantation. Here we show rescue of motility, after transplantation of ENSC, within the *nNOS*^*−/−*^ mouse colon. We further demonstrate robust restoration of nitrergic responses coincident with the development of nNOS^+^ neurons in an nNOS-deficient microenvironment. In addition, we show concurrent rescue of interstitial cells of Cajal (ICC) within the *nNOS*^*−/−*^ colon after ENSC transplantation. Thus, we propose that ENSC can modulate the neuromuscular syncytium via both cell-autonomous and non-cell-autonomous mechanisms to restore function, at the organ level, and ultimately rescue motility.

## Results

### Transplanted ENSC extensively integrate in *nNOS* ^*−/−*^ colon

**Figure 1:**
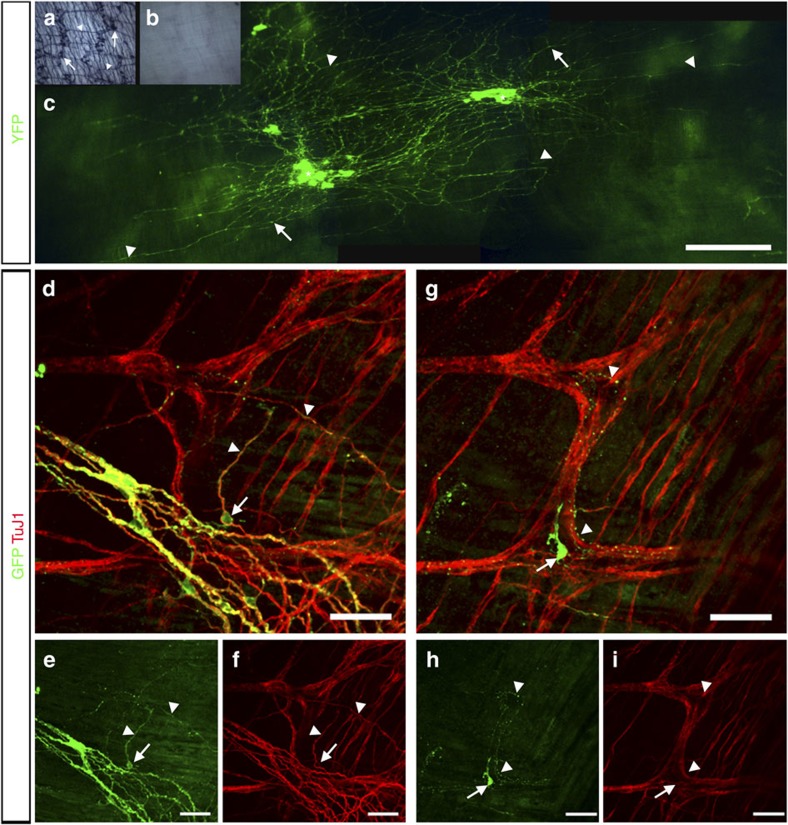
Transplanted ENSC extensively colonize and integrate within the *nNOS*^*−/−*^ mouse colon. (**a**) Representative image of NADPH diaphorase staining in wild-type C57BL/6J. nNOS^+^ cell bodies (arrows), within enteric ganglia, and nNOS^+^ fibres (arrowheads) are indicated. (**b**) Representative image of NADPH diaphorase staining of *nNOS*^*−/−*^ mouse colon demonstrating loss of nitrergic innervation with the absence of NADPH diaphorase staining. (**c**) Representative stereoscopic montage image of transplanted YFP^+^ cells within the *nNOS*^*−/−*^ distal colon at 4 weeks. Transplanted cells form large anastomosing networks comprising cell bodies (arrows) and fibres (arrowheads), including ganglia-like structures (asterisk). Scale bar, 500 μm. (**d**) Example confocal image demonstrating co-expression of the neuronal marker TuJ1 within YFP^+^-transplanted cells. YFP^+^ cell bodies (arrow) project fibres (arrowheads) towards the endogenous neuronal network at the level of the myenteric plexus. (**e**,**f**) Individual channels showing GFP and TuJ1 staining from **d**. (**g**) Representative confocal image demonstrating the presence and integration of transplanted cells within endogenous ganglia. At the level of the myenteric plexus, fine GFP^+^ fibres and varicosities encompass and trace endogenous neuronal fibre tracts (arrowheads). GFP^+^ cells were also identified within ganglia (arrow). (**h**,**i**) Individual channels taken from **g** showing GFP and TuJ1 staining at the level of the myenteric plexus. Scale bar, 50 μm (**d**–**i**).

To isolate ENSC, we used donor *Wnt1*^*cre/+*^*;R26R*^*YFP/YFP*^ mice (P2–P7), in which neural crest cells and their enteric derivatives express endogenous yellow fluorescent protein (YFP). This endogenous expression allowed for isolation and fate-mapping of labelled donor ENSC. Selected YFP^+^ cells maintained expression and formed characteristic neurospheres within 1 month in culture (Supplementary Fig. 1). To assess the composition of neurospheres, immunohistochemistry and qRT–PCR were performed to establish the presence of typical ENS cell types. Such neurospheres were found to express ENS markers such as the pan-neuronal marker TuJ1 (Supplementary Fig. 1a), the neural crest progenitor marker SOX10 (Supplementary Fig. 1b) and the glial marker S100 (Supplementary Fig. 1c). Notably, in addition to multipotent neural crest progenitors, neuronal markers, including NOS^+^ neurons (Supplementary Fig. 1e–g), were observed within neurospheres *in vitro*. Hence, we sought to establish the ability of such neurospheres to colonize and populate *nNOS*^*−/−*^ colon *in vivo.* As opposed to wild-type colon, which contains nNOS^+^ cell bodies and fibres (Fig. 1a), *nNOS*^*−/−*^ mice display complete loss of nNOS^+^ neurons in the colon (Fig. 1b).

We transplanted three YFP^+^ neurospheres (∼6 × 10^4^ cells in total) into the distal colon of *nNOS*^*−/−*^ mice at P14–P17 via laparotomy. Live imaging analysis, 4 weeks after transplantation, revealed the presence of extensive anastomosing networks of transplanted YFP^+^ cells colonizing, on average, 5.46±0.5 mm^2^ (*n*=10) of distal colon at the site of transplantation (Fig. 1c). Subsequent immunohistochemistry revealed more extensive networks of GFP^+^ cells (Supplementary Figs 2 and 3). GFP^+^ filamentous networks could be observed extending in both oral and aboral directions from the site of transplantation (Supplementary Fig. 2 and Supplementary Movie 1) including integration within the proximal colon. Post-acquisition mapping of transplanted cells revealed the largest continuous GFP^+^ network extending 10.79 mm (Supplementary Fig. 2b). Along the length of the colon GFP^+^ cells were found to co-express the neuronal marker TuJ1 (Fig. 1d–f) and project fibres (Fig. 1d, arrowheads), which contacted the endogenous neuronal network at the level of the myenteric plexus. GFP^+^ cells were also identified within endogenous myenteric ganglia (Fig. 1g–i), where fine GFP^+^ fibres and varicosities were observed encompassing and tracing the path of endogenous neuronal fibre tracts (Fig. 1g, arrowheads). Confocal imaging of the entire colon also revealed GFP^+^ cells co-expressing TuJ1 integrated within ganglia along the length of the colon (Supplementary Fig. 3 and Supplementary Movie 2) up to a maximum of 42.4 mm from the site of transplantation thus confirming the ability of transplanted cells to migrate within the *tunica muscularis.* Transplanted cells displayed enteric neuronal characteristics including integration of bipolar (Supplementary Fig. 4a–d) and multipolar GFP^+^ cells (Supplementary Fig. 4e,f) phenocopying the morphology of enteric interneurons and motor neurons, respectively.

### Transplanted ENSC regenerate nNOS^+^ neurons

**Figure 2:**
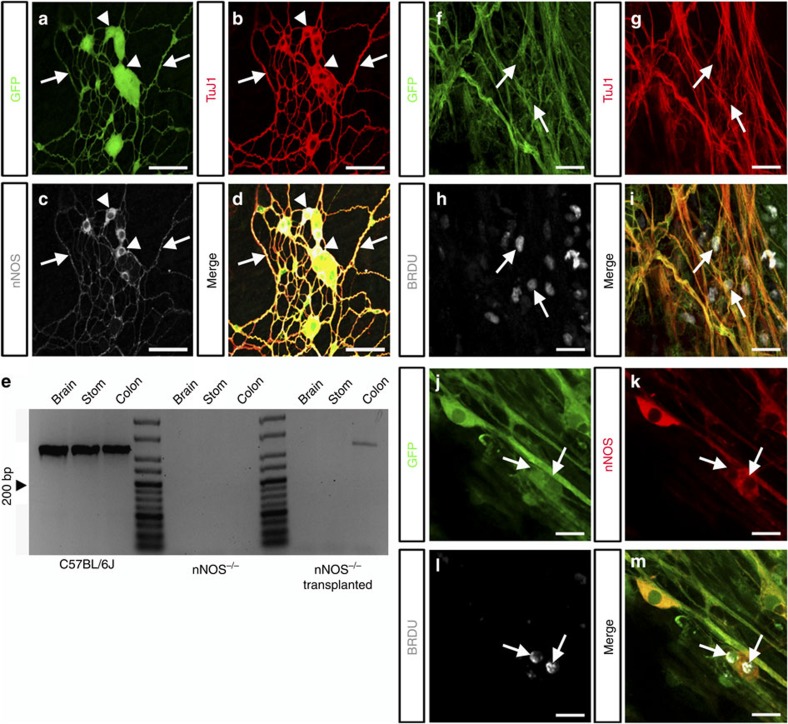
Transplanted ENSC form nNOS^+^ neurons within the *nNOS*^*−/−*^ distal colon. (**a**–**c**) Representative confocal z-stack images demonstrating individual channels for GFP (**a**), TuJ1 (**b**) and nNOS (**c**) within a transplanted cell network present in the *nNOS*^*−/−*^ distal colon. (**d**) Merged image of individual channels shown in (**a**–**c**) demonstrating triple labelling of transplanted YFP^+^ cells (green) co-expressing the neuronal marker TuJ1 (red) and the neuronal nitric oxide synthase marker nNOS (grey). Individual transplanted nNOS^+^ neuronal cell bodies (arrowheads) extend multiple nNOS^+^ projections (arrows) to form an anastomosing network of transplanted cells. Scale bar, 50 μm. (**e**) Representative PCR gel demonstrating specific nNOS expression within transplanted *nNOS*^*−/−*^ colon. (**f**–**m**) Example confocal images of BrdU incorporation within transplanted cells 4 weeks after application. Triple labelled transplanted cells (green, **f**,**j**) co-express either TuJ1 (red, **g**) or nNOS (red, **k**) having incorporated BrdU (grey, **h**,**l**) after administration 24 h post surgery. Scale bar, 20 μm.

To determine if transplanted ENSC have the capacity to develop an NOS^+^ phenotype *in vivo* similar to *in vitro* cultures, immunohistochemistry and RT–PCR were performed. Within the distal colon, transplanted YFP^+^ cells co-expressed both the neuronal marker TuJ1 and the neuronal nitric oxide synthase marker nNOS (Fig. 2a–d). nNOS^+^ neurons were identified within ganglia-like structures (Fig. 2c,d, arrowheads) extending multiple nNOS^+^ projections (Fig. 2c,d, arrows) within the network of transplanted cells. The presence of nNOS^+^ neurons was further confirmed with PCR analysis demonstrating the specific expression of the nNOS transcript within transplanted colon compared with the complete absence of transcript in non-transplanted tissues (Fig. 2e).

To assess the proliferative capacity of transplanted cells, BrdU was applied 24 h post surgery and incorporation was assessed at 4 weeks. Incorporation of BrdU was observed within transplanted cells co-expressing TuJ1 (Fig. 2f–i, arrows) or nNOS (Fig. 2j–m, arrows) suggesting that transplanted ENSC have the ability to proliferate at early post-transplantation stages and subsequently differentiate to form mature neurons including nNOS^+^ neurons within an nNOS-deficient microenvironment.

### Restoration of nitrergic responses in the *nNOS* *^−/−^* colon

**Figure 3:**
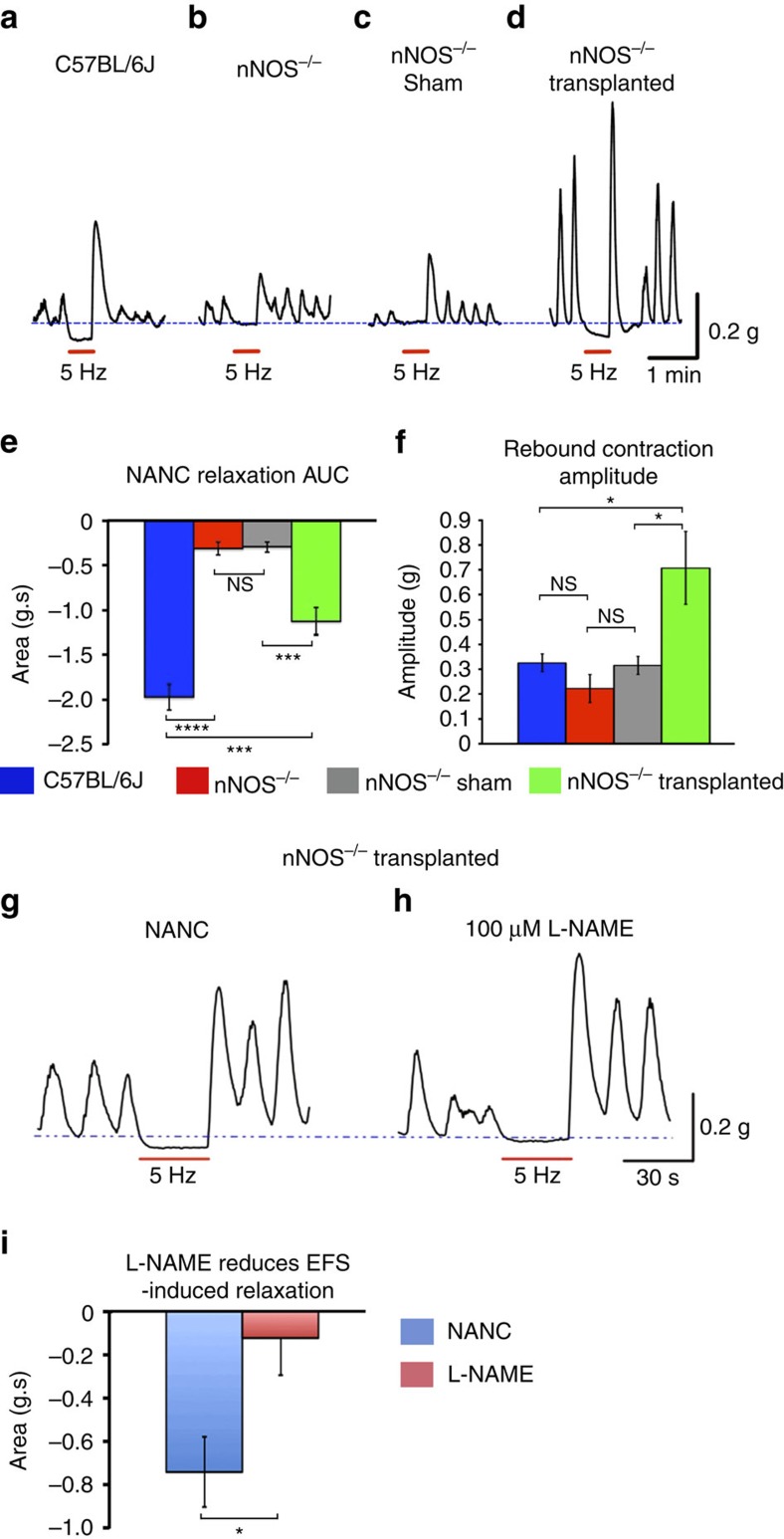
ENSC transplantation restores nitrergic responses in *nNOS*^*−/−*^ distal colon. (**a**–**d**) Representative traces of organ bath contractility in the presence of NANC demonstrating the response, in distal colonic segments, to EFS. (**a**) In response to EFS C57BL/6J distal colon demonstrates nitrergic relaxation, seen as relaxation below basal tension (dotted line). (**b**,**c**) *nNOS*^*−/−*^ non-transplanted and sham-operated animals display a loss of this response. (**d**) Transplanted *nNOS*^*−/−*^ mice display a partial restoration of the response at 4 weeks post transplantation. (**e**,**f**) Quantification of EFS-induced relaxation (area under curve, **e**) and rebound contraction (**f**) in C57BL/6J (blue bars, *n*=5), *nNOS*^*−/−*^ (red bars, *n*=5), sham-operated *nNOS*^*−/−*^ (grey bars, *n*=5) and transplanted *nNOS*^*−/−*^ (green bars, *n*=5) distal colon. ****P≤0.0001, **P≤0.01, *P≤0.05, by Student’s *t*-test. (**g**–**i**) Representative traces of organ bath contractility in transplanted *nNOS*^*−/−*^ colon in the absence or presence of the nitric oxide synthase antagonist L-NAME. (**i**) Summary data of the effect of L-NAME on the EFS-induced nitrergic response in transplanted *nNOS*^*−/−*^ distal colon (*n*=4). *P≤0.05 by Student’s *t*-test. Error bars represent mean±s.e.m. in all panels.

Having demonstrated the ability of ENSC to form nNOS^+^ neurons *in vivo*, we assessed whether this integration could restore nitrergic responses in the *nNOS*^*−/−*^ distal colon. On electrical field stimulation (EFS), C57BL/6J distal colon displayed large EFS-induced relaxations of −1.98±0.15 g s; *n*=5 (Fig. 3a,e). By contrast loss of nNOS within *nNOS*^*−/−*^ mice resulted in significant loss of this relaxatory response (−0.31±0.08 g s; *n*=5, *P*<0.0001, Student’s *t*-test; Fig. 3b,e). Sham-operated *nNOS*^*−/−*^ distal colon displayed similarly abrogated EFS-induced responses (−0.296±0.05 g s; *n*=5; Fig. 3c,e) to control *nNOS*^*−/−*^ distal colon (*P*=0.8376, Student’s *t*-test). Interestingly, after ENSC transplantation there were statistically significant differences in mean values between control *nNOS*^*−/−*^, sham-operated and transplanted *nNOS*^*−/−*^ groups as determined by one-way analysis of variance (ANOVA) (F(2,12)=20.78, *P*≤0.001) with transplanted *nNOS*^*−/−*^ distal colon displaying significant increases in EFS-induced relaxation (−1.13±0.16 g s; *n*=5; Fig. 3d,e) compared to sham-operated *nNOS*^*−/−*^ mice (*P*=0.0012, Student’s *t*-test). There was, however, a statistical difference between C57BL/6J distal colon and that of transplanted *nNOS*^*−/−*^ mice (*P*=0.0034, Student’s *t*-test). On EFS, C57BL/6J distal colon also displayed post-stimulation rebound contractions with a mean amplitude of 0.326±0.04 g; *n*=5 (Fig. 3a,f). Similarly, *nNOS*^*−/−*^ mice displayed these post-stimulation rebound contractions (0.22±0.06 g; *n*=5, *P*=0.1586, Student’s *t*-test; Fig. 3b,f). Sham-operated *nNOS*^*−/−*^ distal colon displayed similar responses (0.315±0.04 g; *n*=5; Fig. 3c,f) to *nNOS*^*−/−*^ mice (*P*=0.2083, Student’s *t*-test). Interestingly, after ENSC transplantation, there were statistically significant differences in mean values between control *nNOS*^*−/−*^, sham-operated and transplanted *nNOS*^*−/−*^ groups by one-way ANOVA (F(2,12)=7.61, *P*=0.007) with transplanted *nNOS*^*−/−*^ distal colon displaying significant increases in rebound contraction amplitude (0.708±0.15 g; *n*=5; Fig. 3d,f) compared to sham-operated *nNOS*^*−/−*^ mice (*P*=0.032, Student’s *t*-test) and C57BL/6J distal colon (*P*=0.0354, Student’s *t*-test).

To establish if the restored relaxatory response was due to the presence of transplanted nNOS^+^ neurons, EFS-induced responses observed in non-adrenergic non-cholinergic conditions were analysed in the presence and absence of the nitric oxide synthase blocker L-NAME. L-NAME significantly reduced this EFS-induced relaxation (−0.74±0.17 g s versus −0.12±0.16 g s; *n*=4; *P*=0.0389, Student’s *t*-test) in transplanted *nNOS*^*−/−*^ colon (Fig. 3g–i). Taken together, these results indicate that transplantation of ENSC and development of nNOS^+^ neurons in the *nNOS*^*−/−*^ distal colon results in partial restoration of nitrergic responses.

### ENSC transplantation increases basal contractile properties

Organ bath physiology also revealed large amplitude basal contractions in transplanted *nNOS*^*−/−*^ distal colonic segments (Fig. 3d). To investigate these responses, basal contractile patterns were recorded in both the distal and proximal colonic segments in control conditions (Krebs solution), and after the addition of individual neurotransmitter antagonists or tetrodotoxin (TTX).

**Figure 4:**
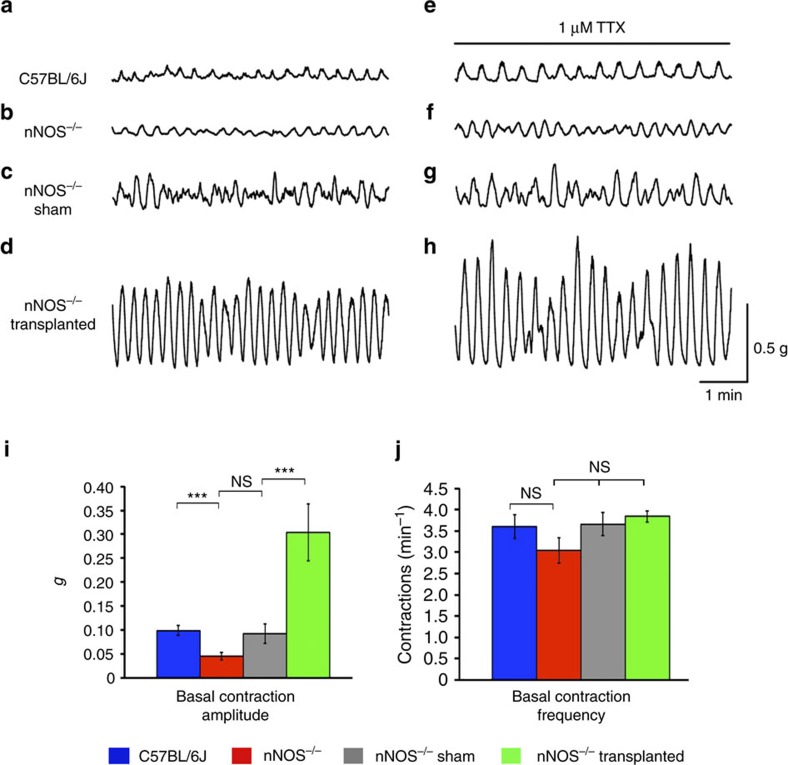
ENSC transplantation alters basal contractile patterns in *nNOS*^*−/−*^ distal colon. (**a**–**d**) Representative organ bath contractility traces demonstrating basal contractile properties of C57BL/6J, *nNOS*^*−/−*^, sham-operated *nNOS*^*−/−*^ and transplanted *nNOS*^*−/−*^ distal colon in control conditions. (**e**–**h**) Representative organ bath contractility traces demonstrating basal contractile properties of C57BL/6J, *nNOS*^*−/−*^, sham-operated *nNOS*^*−/−*^ and transplanted *nNOS*^*−/−*^ distal colon from **a**–**d** after the addition of TTX. (**i**) Quantification of basal contractile amplitude in C57BL/6J (blue bars), *nNOS*^*−/−*^ (red bars), sham-operated *nNOS*^*−/−*^ (grey bars) and transplanted *nNOS*^*−/−*^ (green bars) distal colon. *n*=5 for each group. ***P≤0.001 by Student’s *t*-test. (**j**) Quantification of basal contractile frequency in C57BL/6J (blue bars), *nNOS*^*−/−*^ (red bars), sham-operated *nNOS*^*−/−*^ (grey bars) and transplanted *nNOS*^*−/−*^ (green bars) distal colon. *n*=5 for each group. Error bars represent mean±s.e.m. in all panels.

Wild-type C57BL/6J distal colon displayed significantly larger amplitude basal contractions (0.10±0.01 g; *n*=5) compared to *nNOS*^*−/−*^ mice (0.05±0.01 g; *n*=5; *P*=0.0029, Student’s *t*-test; Fig. 4a,b,i). The distal colon of sham-operated *nNOS*^*−/−*^ mice was found to display similar contractions (0.09±0.02 g; *n*=5; Fig. 4c,i) to that of control *nNOS*^*−/−*^ mice (*P*=0.0592, Student’s *t*-test). One-way ANOVA analysis suggested that there were statistically significant differences in basal contractile amplitude (F(2,12)=14.43, *P*=0.001) between control *nNOS*^*−/−*^, sham-operated and transplanted *nNOS*^*−/−*^ mice with significant increases in the average contractile amplitude in transplanted *nNOS*^*−/−*^ distal colon (0.30±0.06 g, *n*=5) compared with sham-operated *nNOS*^*−/−*^ (*P*=0.0096, Student’s *t*-test; Fig. 4c,d,i). Contractile frequency, however, did not appear to be affected as determined by one-way ANOVA (F(2,12)=2.96, *P*=0.090, Fig. 4j). In addition, these increased basal contractile properties were not significantly affected by addition of individual neural antagonists or TTX (Fig. 4e–h and Supplementary Fig. 6) suggesting that transplantation of ENSC can lead to potential changes in underlying myogenic motility patterns.

To establish if ENSC transplantation can alter motility patterns beyond the transplanted distal colonic region, proximal colonic segments were investigated for changes in basal contractile patterns. Similar to the distal colon, wild-type C57BL/6J proximal colon displayed significantly larger amplitude contractions (0.34±0.04 g; *n*=3) compared to control *nNOS*^*−/−*^ mice (0.10±0.02 g; *n*=3; *P*=0.0044, Student’s *t*-test; Supplementary Fig. 5a,b,i). Contractile amplitude in the proximal colon of sham-operated *nNOS*^*−/−*^ mice was similar (0.10±0.03 g, *n*=3; *P*=0.9931, Student’s *t*-test) to that of *nNOS*^*−/−*^ mice (Supplementary Fig. 5b,c,i). One-way ANOVA analysis suggested that there were statistically significant differences in basal contractile amplitude (F(2,6)=7.38, *P*=0.024) between control *nNOS*^*−/−*^, sham-operated and transplanted *nNOS*^*−/−*^ group means with transplanted *nNOS*^*−/−*^ proximal colon displaying contractile events with significantly increased amplitude (0.32±0.07 g; *n*=3) compared with sham-operated *nNOS*^*−/−*^ mice (*P*=0.0412, Student’s *t*-test; Supplementary Fig. 5c,d,i). Contractile frequency, however, did not appear to be affected as determined by one-way ANOVA (F(2,12)=1.21, *P*=0.362; Supplementary Fig. 5j). Thus, we conclude that ENSC transplantation to the distal colon results in changes in colonic motor patterns at sites distant from the transplanted region. Again, similar to the distal colon, these increased basal contractions were not affected by TTX (Supplementary Fig. 5e–h and Supplementary Fig. 6). The failure of TTX to attenuate these large amplitude colonic contractions in both the distal and proximal colon suggests that non-cell-autonomous changes in the neuromusculature occur post transplant.

### Transplantation decreases total intestinal transit time

**Figure 5:**
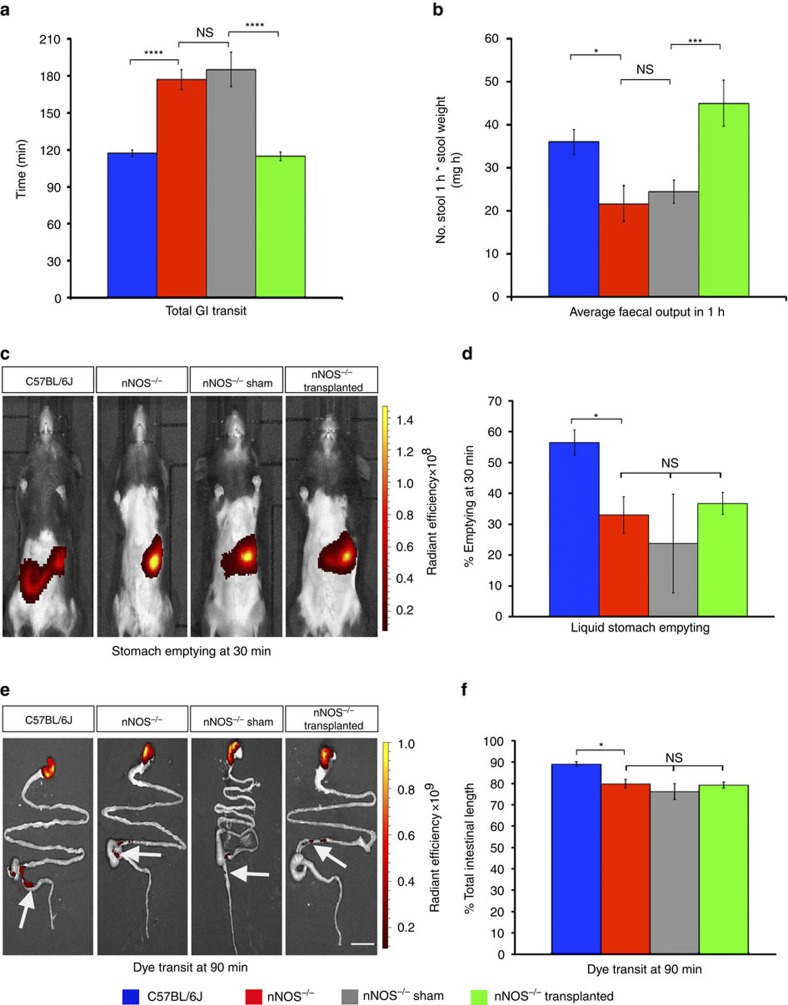
Analysis of GI transit time and stool production in transplanted *nNOS*^*−/−*^ mice. (**a**,**b**) Summary data of total GI transit time (**a**) measured after oral gavage of dye, and faecal output (**b**), 4 weeks after transplantation in C57BL/6J (blue bars), non-transplanted *nNOS*^*−/−*^ (red bars), sham-operated *nNOS*^*−/−*^ (grey bars) and transplanted *nNOS*^*−/−*^ (green bars) mice. *n*=5 for each group, ****P≤0.0001, ***P≤0.001, **P≤0.01, *P≤0.05 by Student’s *t*-test. (**c**) Representative *in vivo* fluorescent images 30 min after gavage of a fluorescent marker. (**d**) Quantification of percentage stomach emptying at 30 min in C57BL/6J (blue bars), *nNOS*^*−/−*^ (red bars), sham-operated *nNOS*^*−/−*^ (grey bars) and transplanted *nNOS*^*−/−*^ (green bars) mice. *n*=3 for each group. *P≤0.05 by Student’s *t*-test. (**e**) Example fluorescent images of the C57BL/6J, *nNOS*^*−/−*^, sham-operated *nNOS*^*−/−*^ and transplanted *nNOS*^*−/−*^ intestine *ex vivo*, 90 min after gavage of a fluorescent marker. (**f**) Summary data of dye transit at 90 min in C57BL/6J (blue bars), *nNOS*^*−/−*^ (red bars), sham-operated *nNOS*^*−/−*^ (grey bars) and transplanted *nNOS*^*−/−*^ (green bars) mice. *n*=3 for each group. *P≤0.05 by Student’s *t*-test. Error bars represent mean±s.e.m. in all panels.

Previous studies have identified motility issues, including slow transit, in the colon of *nNOS*^*−/−*^ mice. To assess the impact of ENSC transplantation, including the effect restored nitrergic responses and increased basal contractile properties would have on motility, total gastrointestinal (GI) transit time was measured. Control *nNOS*^*−/−*^ mice displayed significantly prolonged transit time (177±8.15 min; *n*=5) compared to C57BL/6J mice (117.4±2.64 min; *n*=5; *P*=0.0001, Student’s *t*-test; Fig. 5a). Sham-operated *nNOS*^*−/−*^ mice displayed similar transit time (185.2±14.08 min; *n*=5) to non-transplanted *nNOS*^*−/−*^ controls (*P*=0.63, Student’s *t*-test). Notably, there were significant differences between control *nNOS*^*−/−*^, sham-operated and transplanted *nNOS*^*−/−*^ group means as determined by one-way ANOVA (F(2,12)=16.02, *P*<0.0001). Moreover, total GI transit time in transplanted *nNOS*^*−/−*^ mice was significantly decreased (114.8±3.60 min; *n*=5; *P*=0.0001, Student’s *t*-test) compared with sham-operated *nNOS*^*−/−*^ mice, reducing transit time towards levels observed in C57BL/6J mice (*P*=0.5761, Student’s *t*-test) suggesting rescue of motility (Fig. 5a).

Analysis of faecal output revealed a significant reduction in output in *nNOS*^*−/−*^ mice (21.61±3.71 mg h^−1^; *n*=5) compared with C57BL/6J mice (35.97±2.63 mg h^−1^; *n*=5; *P*=0.0134, Student’s *t*-test; Fig. 5b). In addition, sham-operated *nNOS*^*−/−*^ displayed similar faecal output (24.41±2.41 mg h^−1^; *n*=5) to that of *nNOS*^*−/−*^ controls (*P*=0.544, Student’s *t*-test). Interestingly, there were statistically significant differences in faecal output between control *nNOS*^*−/−*^, sham-operated and transplanted *nNOS*^*−/−*^ group means as determined by one-way ANOVA (F(2,12)=11.51, *P*=0.002) with transplantation of ENSC resulting in significant increases in faecal output in transplanted *nNOS*^*−/−*^ mice (44.95±4.77 mg h^−1^; *n*=5) compared with sham-operated *nNOS*^*−/−*^ mice (*P*=0.0049, Student’s *t*-test), restoring faecal output to levels comparable to C57BL/6J mice (*P*=0.1378, Student’s *t*-test).

### Upper GI transit is not affected by ENSC transplantation

As *nNOS*^*−/−*^ mice display pan-enteric deficits in nNOS signalling and have been shown to have delayed gastric emptying, we sought to assess if transplantation to the distal colon could affect intestinal transit parameters outside of the colonic region. Using fluorescent *in viv*o imaging, liquid stomach emptying at 30 min (Fig. 5c,d) was significantly delayed in *nNOS*^*−/−*^ mice (32.9±5.9%, *n*=3) compared to C57BL/6J mice (56.4±4.6%; *n*=3; *P*=0.0316, Student’s *t*-test) similar to previously described studies. Notably, no difference was observed in liquid stomach emptying between control *nNOS*^*−/−*^, sham-operated and transplanted *nNOS*^*−/−*^ group means as determined by one-way ANOVA (F(2,6)=1.00, *P*=0.421; Fig. 5c,d).

To assess partial intestinal transit, mice were killed 90 min after gavage of a fluorescent dye. Fluorescent imaging was performed *ex vivo* and the distance the dye had transited was calculated as a percentage of total intestinal length (Fig. 5e,f). *nNOS*^*−/−*^ mice displayed reduced intestinal transit (79.9±2.1%, *n*=3) compared to C57BL/6J mice (89.1±1.1%, *n*=3; *P*=0.0176, Student’s *t*-test). Again, there was no difference between control *nNOS*^*−/−*^, sham-operated and transplanted *nNOS*^*−/−*^ transit distance at 90 min as determined by one-way ANOVA (F(2,6)=0.56, *P*=0.6). We conclude that as both stomach emptying and partial intestinal transit time are unaffected by transplantation, the overall improvement in total intestinal transit time in transplanted *nNOS*^*−/−*^ mice is due to substantial increases in colonic transit.

### ENSC transplantation restores ICC numbers

We next sought to ensure that the changes in GI function following transplantation were not secondary to other phenomena such as inflammation. On initial dissection, and after careful analysis, no significant changes in the gross anatomy of the colon or evidence of inflammatory responses were observed (Supplementary Fig. 7a). In addition, no inflammation was observed on histological examination (Supplementary Fig. 7b). Histological analysis also revealed that there were no differences in either colonic diameter (*P*>0.05; Supplementary Fig. 7c,e) or muscle thickness (*P*>0.05; Supplementary Fig. 7d,f) when comparing both the proximal and distal colon in *nNOS*^*−/−*^, sham-operated *nNOS*^*−/−*^ and transplanted *nNOS*^*−/−*^ animals by one-way ANOVA.

**Figure 6:**
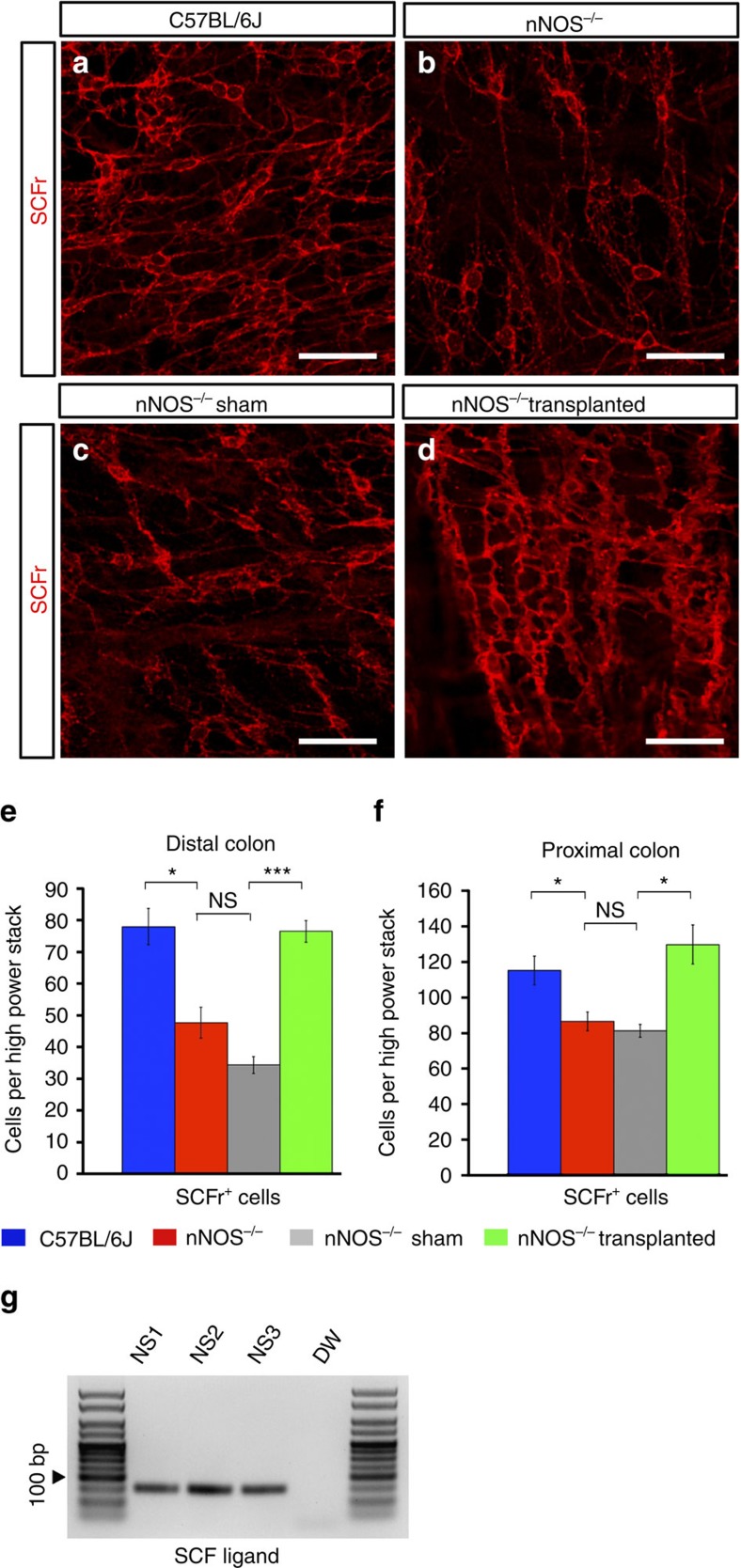
ENSC transplantation alters ICC networks in *nNOS*^*−/−*^ colon. (**a**–**d**) Representative z-stack confocal images of ICC in C57BL/6J, *nNOS*^*−/−*^, sham-operated *nNOS*^*−/−*^ and transplanted *nNOS*^*−/−*^ distal colon. Scale bar, 50 μm. (**e**,**f**) Quantification of ICC numbers in the distal (**e**) and proximal (**f**) colon in C57BL/6J (blue bars), *nNOS*^*−/−*^ (red bars), sham-operated *nNOS*^*−/−*^ (grey bars) and transplanted *nNOS*^*−/−*^ (green bars) mice calculated as a mean of five high-power stacks per region per mouse. *n*=3 for each group. ***P≤0.001, *P*≤0.05 by Student’s *t*-test. Error bars represent mean±s.e.m. in all panels. (**g**) Representative PCR gel demonstrating expression of SCF ligand in *Wnt1*^*cre/+*^*;R26R*^*YFP/YFP*^-derived neurospheres (NS). Neurosphere cultures were derived from separate *Wnt1*^*cre/+*^*;R26R*^*YFP/YFP*^ intestines (*n*=3). Autoclaved Milli-Q water (DW) was used as a non-template negative control.

To assess if ENSC transplantation may alter other cells involved in neuromuscular signalling and excitation–contraction coupling, the numbers of ICC were quantified in both the distal (Fig. 6a–e) and proximal (Fig. 6f and Supplementary Fig. 8) colon. Notably, ICC were reduced in both distal (48±5; *n*=3; Fig. 6e) and proximal (87±5; *n*=3; Fig. 6f) *nNOS*^*−/−*^ colonic segments compared to C57BL/6J distal (78±6; *n*=3; *P*=0.0158, Student’s *t*-test) or proximal colon (115±8; *n*=3; *P*=0.0410, Student’s *t*-test). No difference was found between ICC numbers in either the distal (34±3; *n*=3; *P*=0.0743, Student’s *t*-test) and proximal colon (81±4; *n*=3; *P*=0.4504, Student’s *t*-test) in sham-operated *nNOS*^*−/−*^ compared to control *nNOS*^*−/−*^ mice (Fig. 6e,f). By contrast, one-way ANOVA analysis determined that there were statistically significant differences in ICC numbers in both the distal (F(2,6)=6.47, *P*=0.032) and proximal (F(2,6)=26.29, *P*=0.001) colon when comparing non-transplanted *nNOS*^*−/−*^, sham-operated *nNOS*^*−/−*^ and transplanted *nNOS*^*−/−*^ mice. Of interest, increased ICC numbers were observed in transplanted *nNOS*^*−/−*^ mice both in the distal (77±3; *n*=3) and proximal colon (130±11; *n*=3) compared to sham-operated *nNOS*^*−/−*^ distal (*P*=0.0006, Student’s *t*-test; Fig. 6e) and proximal (*P*=0.0135, Student’s *t*-test; Fig. 6f) colon, respectively. This increase in ICC numbers associated with ENSC transplantation suggests indirect modulation of cells that contribute to the neuromuscular syncytium, alongside the integration of transplanted neural crest-derived cells. To further assess this indirect modulation, we investigated if ENSC express trophic factors that may influence ICC development. PCR analysis demonstrated the expression of the stem cell factor (SCF) ligand within ENSC neurospheres *in vitro* (Fig. 6g) providing a possible mechanism by which transplanted cells could alter ICC development and maintenance after transplantation.

## Discussion

Recent studies have demonstrated the functional integration of both mouse and human ENSC-derived neurons in wild-type mouse colon after *in vivo* transplantation^16^,^17^,^18^. Functional analysis in these animals that lack a neuropathological phenotype is restricted to individual or groups of transplanted neurons rather than assessment of colonic physiology. We now report that transplantation of a population of selected enteric neural crest-derived cells has a clear and positive functional impact, rescuing motility in a pathophysiological mouse model that recapitulates the phenotype of several clinically relevant human disorders. We demonstrate that this rescue is achieved through both cell-autonomous restoration of nitrergic responses that were absent, and non-cell-autonomous rescue of ICC numbers, which were also found to be deficient in the *nNOS*^*−/−*^ colon.

The ability to form nitrergic neurons is a critical step in the development of ‘normal’ enteric circuitry and many enteric disorders would likely benefit from the transplantation and engraftment of nNOS^+^ cells. In clearly demonstrating the potential to restore nNOS neurons after ENSC transplantation, which to our knowledge is the first study to show functional effects at the organ level, we believe ENSC therapies could impact widely at the clinical level. Interestingly, transplantation of ENSC led to restoration of nitrergic responses and increases in post-stimulation ‘rebound’ contraction. Previous studies have suggested that this post-stimulation contraction is mediated via nitric oxide^33^ or through a generalized inhibitory response via eicosanoids rather than linked to a distinct inhibitory transmitter^34^. The finding that transplantation of ENSC cells restored nitrergic inhibitory responses and altered post-stimulus responses suggests adaptation of neuromuscular signalling mechanisms at multiple levels. Of note, there are differences in the methodology of our study and that of these earlier studies, hence further investigation is required to assess the functional interaction of transplanted ENSC at the cellular level. Furthermore, the novel finding that ENSC transplantation rescues ICC numbers in the *nNOS*^*−/−*^ colon in a non-cell-autonomous fashion raises interesting questions regarding the interaction of transplanted ENSC with associated cells within intestinal tissues. Intestinal excitation–contraction coupling is extremely complex, with GI smooth muscle receiving inputs from multiple excitable cells. ICC exist within extensive networks throughout the intestinal tract^35^ exhibiting pacemaker activity^36^,^37^,^38^ and transducing neural signals to the smooth muscle^39^,^40^. Recent studies show that ICC are innervated by nitrergic nerves^41^ and express nitric oxide sensitive guanylate cyclase in both guinea pig^42^ and mouse colon^43^ and that disruptions in nNOS signalling can result in loss of ICC in the stomach^25^,^44^. The findings of similar disruptions in ICC networks, in the present study, along the length of the *nNOS*^*−/−*^ colon further emphasize the link between NOS signalling and ICC development. Our previous work has demonstrated that the selection of ENSC, as in this study, excludes incorporation of mesenchyme-derived ICC^45^. Notably, previous studies have suggested that enteric neurons are the dominant source of SCF, the natural ligand for c-Kit^46^, and that 60% of nNOS^+^ enteric neurons express SCF^47^ possibly providing a direct signalling link between enteric neurons and ICC. The finding that ENSC-derived neurospheres express SCF ligand in line with these earlier reports provides a possible mechanism whereby ICC modification post transplantation is likely through SCF signalling from transplanted neurons.

One of the challenges for cell replenishment therapies is scaling up for potential human application. It remains possible that significant cell numbers will be required to facilitate functional outcomes in human patients. A recent study has demonstrated derivation of enteric neural crest from human pluripotent stem cells and potential rescue of a Hirschsprung phenotype^48^. As opposed to our study, this investigation transplanted up to 4 million cells to *Ednrb*^*s−l/s−l*^ (SSL/LEJ) colon. While transplantation led to survival of mice, no mechanism was presented of the graft-mediated host rescue. The findings of our study suggest that in addition to engraftment of neural crest cells, non-cell-autonomous modification of the neuromuscular apparatus may be responsible for this rescue. Our study thus highlights the potential to apply a limited number of cells, to a particular area, which subsequently could impact on function throughout the organ and have significant clinical benefits. In addition, we demonstrate the potential for collecting postnatal tissue for use in transplantation studies and the ability of cells from this source to restore function. This caveat has significant therapeutic benefits as ENSC could be collected autologously or from matched donors to limit the potential for immunological rejection. Moreover, previous long-term safety studies using identical postnatal ENSC have demonstrated long-term survival of ENSC-derived cells restricted only to the region of transplantation^16^, which may provide a substantial benefit over the potential therapeutic application of cells derived from pluripotent sources.

We conclude that this study provides the first evidence that ENSC can rescue GI motility within a neuropathic model and may provide the basis for development of targeted cellular therapies for enteric neuropathies.

## Methods

### Animals

Male and female *Wnt1*^*cre/+*^*;R26R*^*YFP/YFP*^ mice, in which neural crest cells express YFP, were used as donors to obtain YFP^+^ ENSC. Heterozygote nNOS (B6.129S4-*Nos1*^*tm1Plh*^/J) mice were obtained from The Jackson Laboratory (Bar Harbor, MN, USA). Male and female homozygote nNOS knockout (*nNOS*^*−/−*^) mice were bred and maintained for use as recipients. Four–5-week-old C57BL/6J mice were obtained from The Jackson Laboratory (Bar Harbor, MN, USA) and killed as age-matched controls at 6 weeks. Animals used for these studies were maintained, and the experiments performed, in accordance with the UK Animals (Scientific Procedures) Act 1986 and approved by the University College London Biological Services Ethical Review Process. Animal husbandry at UCL Biological Services was in accordance with the UK Home Office Certificate of Designation.

### Cell isolation and enrichment

The entire small intestine and colon was obtained from early postnatal (P2–P7) *Wnt1*^*cre/+*^*;R26R*^*YFP/YFP*^ mice after cervical dislocation, and removed to sterile PBS for further dissection. Jejunum, ileum and colon muscle strips were obtained following removal of the mucosa via fine dissection. Intestinal cells were dissociated and YFP^+^ cells isolated using fluorescence-activated cell sorting with a MoFloXDP cell sorter (Beckman Coulter, UK). YFP positive (YFP^+^) cells were selected using a 530/40 filter set. Gating parameters were set using cells from wild-type gut and applied to increase specificity of selection of YFP^+^ cells.

### Neurosphere culture

YFP^+^ cells were plated on fibronectin-coated six-well dishes in ‘neurosphere medium’ (NSM; DMEM F12 supplemented with B27 (Invitrogen, UK), N2 (Invitrogen, UK), 20 ng ml^−1^ EGF (Peprotech, UK), 20 ng ml^−1^ FGF (Peprotech, UK) and Primocin antibiotic (InvivoGen, UK) and maintained in culture for up to 4 weeks. Typically such cultures from early postnatal (P2–P7) intestine formed ‘neurospheres’ at ∼1 week in culture.

### *In vivo* ENSC transplantation

YFP-expressing neurospheres derived from *Wnt1*^*cre/+*^*;R26R*^*YFP/YFP*^ mice were transplanted into the distal colon of P14–P17 *nNOS*^*−/−*^, via laparotomy under isoflurane anaesthetic. Briefly, the distal colon was exposed and a small pocket was created in the *tunica muscularis* with the bevel of a 30G needle. A neurosphere, containing ∼2 × 10^4^ YFP^+^ cells, was subsequently transplanted to this site by mouth pipette using a pulled glass micropipette. Each transplanted tissue typically received three neurospheres (∼6 × 10^4^ YFP^+^ cells in total). Transplanted *nNOS*^*−/−*^ mice were typically maintained for 4 weeks post transplantation before killing and removal of the colon for analysis. ‘Sham’ operations were performed as controls in which the intestine was manipulated in an identical fashion without the addition of YFP^+^ cells.

### RT–PCR

Total RNA was isolated from C57BL/6J, *nNOS*^*−/−*^ or transplanted *nNOS*^*−/−*^ brain, stomach and colon using TRIzol reagent (Life Technologies Ltd, Paisley, UK) and treated with DNase I (Qiagen, Manchester, UK). First-strand cDNA was amplified from 1 μg RNA using SuperScript VILO cDNA Synthesis Kit (Life Technologies Ltd, Paisley, UK). PCR was performed using region-specific primers for *nNOS* (Supplementary Table 1) using HotStarTaq DNA Polymerase (Qiagen, Manchester, UK). PCR reactions were performed in a PTC-200 Peltier Thermal Cycler (MJ Research Inc. Waltham, MA, USA). The amplification profile was 95 °C for 3 min, 35 cycles of 94 °C for 30 s, 60 °C for 60 s and 72 °C for 30 s, followed by a final step of 72 °C for 2 min. RT–PCR amplification fragments were analysed on a 2% agarose gel alongside a Hyperladder 25 bp marker (Bioline, London, UK).

### qRT–PCR

Total RNA was isolated from three pooled neurospheres at the time of surgery using an RNeasy Micro Kit (Qiagen, Hilden, Germany), according to the manufacturer’s instructions. First-strand cDNA was amplified from 100 ng RNA using SuperScript VILO cDNA Synthesis Kit (Life Technologies Ltd, Paisley, UK). RT quantitative PCR was performed with an ABI Prism 7500 sequence detection system (Applied Biosystems) using the Quantitect SYBR Green PCR kit (Qiagen, Hilden, Germany) according to the manufacturer’s instructions. qRT–PCR was performed in triplicate using region-specific primers for GAPDH, TuJ1, SOX10 and S100 (Supplementary Table 1). Gene expression data were expressed as a proportion of GAPDH housekeeping gene, as a reference, using a 1/ΔCt calculation.

### Immunohistochemistry

Neurosphere immunohistochemistry was performed following paraformaldehyde fixation (4% w/v in 0.1 mol l^*−*1^ PBS for 45 min at 22 °C). After fixation, neurospheres were washed for 1 h in PBS (0.01 mol l^*−*1^, pH 7.2 at 4 °C). Neurospheres were blocked for 1 h (0.1 mol l^*−*1^ PBS containing 1% Triton X-100, 1% BSA and 0.15% glycine) at 22 °C. The primary antibodies used in the study are listed in Supplementary Table 2. Intact neurospheres were incubated in primary antibody (diluted in 0.1 mol l^*−*1^ PBS containing 1% Triton X-100, 1% BSA and 0.15% glycine) overnight at 4 °C and immunoreactivity was detected using secondary antibodies (1:500 in 0.1 mol l^*−*1^ PBS, 1 h at room temperature, Supplementary Table 3). Before mounting, neurospheres were washed thoroughly in PBS (0.1 mol l^*−*1^ PBS for 1 h at 22 °C).

Whole-mount immunohistochemistry was performed on transplanted colonic segments after excision and removal of the mucosa by sharp dissection. Tissues were fixed in paraformaldehyde (4% w/v in 0.1 mol l^*−*1^ PBS for 45 min at 22 °C). After fixation, tissues were washed for 24 h in PBS (0.01 mol l^*−*1^, pH 7.2 at 4 °C). Tissues were blocked for 1 h (0.1 mol l^*−*1^ PBS containing 1% Triton X-100, 10% sheep serum). Tissues were incubated in primary antibody (diluted in 0.1 mol l^*−*1^ PBS containing 1% Triton X-100, 10% sheep serum, Supplementary Table 2) for 48 h at 4 °C and immunoreactivity was detected using the secondary antibodies listed in Supplementary Table 3 (1:500 in 0.1 mol l^*−*1^ PBS, 1 h at room temperature). Before mounting, tissues were washed thoroughly in PBS (0.1 mol l^*−*1^ PBS for 2 h at 22 °C). Control tissues were prepared by omitting primary or secondary antibodies. Tissues and neurospheres were examined using a LSM710 meta confocal microscope (Zeiss, Germany). Confocal micrographs were digital composites of the Z-series of scans (0.5 μm optical sections). For cell-counting experiments, five Z-series scans (21 × 1 μm optical sections including longitudinal muscle, myenteric plexus and circular muscle layers) were obtained in each region per animal. Z-series files were blinded before counting. Individual cells were identified with DAPI co-labelling and cell counts performed across each individual z-scan using the cell counter plugin (FIJI). Each Z-series count (21 sections) was summed and average cell number was calculated as a mean of five Z-series scans per region in each animal. For confocal montage experiments, tissues were examined using a LSM880 multiphoton microscope (Zeiss, Germany). Confocal micrographs of whole mounts were digital composites of the Z-series of scans stitched using Zen software (Zeiss, Germany). Final images were constructed using FIJI software^49^ after applying a post-acquisition Gaussian filter with a *σ* of 2.

For NADPH diaphorase staining, colonic tissues were prepared, fixed and washed as above. NADPH diaphorase activity was detected by incubating tissues in 0.1 mol l^*−*1^ PBS containing 0.05% Triton X-100, 1 mg ml^−1^ β-NADPH (Sigma, UK) and 0.5 mg ml^−1^ nitrobluetetrazolium (Sigma, UK) for 20 min at 37 °C. After staining, the tissues were washed thoroughly in PBS (0.1 mol l^*−*1^) before mounting.

Haemeotoxylin and eosin colonic cryostat sections (20 μm) were obtained from frozen gelatin-embedded samples using a Leica CM1900 UV Cryostat (Leica Microsystems, UK) and processed for haemeotoxylin and eosin. Briefly, frozen colonic sections were post fixed in PFA (4% w/v in 0.1 mol l^*−*1^ PBS for 45 min at 22 °C), washed thoroughly and haemeotoxylin solution (Harris modified; Sigma, UK) applied for 7 min at 22 °C. After washing, slides were immersed in acid alcohol (1% HCl in 70% EtOH) for 10 s and washed before application of 1% eosin Y (Fisher Scientific, UK) for 5 min at 22 °C. Samples were subsequently washed, dehydrated and cleared in Histoclear (2 × 1 min at 4 °C; National Diagnostics, UK) before mounting.

### *In vivo* transit analysis

To test GI transit, either 100 μl Gastrosense 750 (Perkin Elmer, USA) or 100 μl Brilliant Blue FCT (E122) solution (Langdales, UK) was administered to the stomach via gavage at 6 weeks (4 weeks post transplantation). Total GI transit time was calculated from time of administration to the first visualization of dye in the stool. Stool output in 1 h was calculated as: total number of stool in 1 h × stool weight. To assess stomach emptying and partial transit time, Gastrosense 750 fluorescence was imaged using an IVIS Lumina III *In Vivo* Imaging System (Perkin Elmer, USA). At 30 min, *in vivo* images were obtained and percentage stomach emptying calculated as: 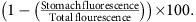


To assess partial intestinal transit, the GI tract (stomach to terminal colon) was removed 90 min after Gastrosense 750 administration. Flourescence images were obtained of the GI tract and percentage intestinal transit calculated as: 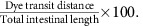


### Contractility

Longitudinal colonic muscle strips were isolated and the mucosa removed by sharp dissection in oxygenated Krebs solution. Longitudinal muscle strips were mounted in tissue baths (10 ml, SI-MB4; World Precision Instruments Ltd, UK) connected via suture to force transducers (SI-KG20, World Precision Instruments Ltd, UK) under an initial tension of 0.5 g. Tissues were maintained at 37 °C with perfusion of oxygenated Krebs solution. Following a 60 min equilibration period, activity was recorded using a Lab-Trax-4 data acquisition system (World Precision Instruments Ltd, UK) in the absence and presence of non-adrenergic non-cholinergic conditions (Atropine; 1 μM, Phentolamine hydrochloride; 1 μM, Propranolol hydrochloride; 1 μM). Nerves were stimulated for 30 s (5 Hz; 40 V; 0.3 ms pulse duration) via EFS via platinum electrode loops placed at each end of the muscle strip using a MultiStim System (D330, World Precision Instruments Ltd, UK). Addition of L-NAME (100 μM) or TTX (1 μM) to the bath solution was used to assess the nitrergic and neurally mediated responses, respectively. EFS gave rise to neural responses that were sensitive to TTX. Data were collected, stored and analysed by computer using a data acquisition programme (Labscribe 2-1, World Precision Instruments Ltd, UK).

### Statistical analysis

Data are expressed as mean±s.e.m. Differences in the data were evaluated between *nNOS*^*−/−*^ control, sham-operated and transplanted groups using one-way ANOVA and subsequent intergroup differences were determined by unpaired Student’s *t*-test. *P* values <0.05 were taken as statistically significant. The ‘*n* values’ reported refer to the number of mice or colonic segments used for each protocol. Each muscle was taken from a separate animal.

### Data availability

The authors declare that all data supporting the findings of this study are available within the article and its Supplementary Information files or from the corresponding author on reasonable request.

## Additional information

**How to cite this article:** McCann, C. J. *et al*. Transplantation of enteric nervous system stem cells rescues nitric oxide synthase deficient mouse colon. *Nat. Commun.* **8**, 15937 doi: 10.1038/ncomms15937 (2017).

**Publisher’s note:** Springer Nature remains neutral with regard to jurisdictional claims in published maps and institutional affiliations.

## Supplementary Material

Supplementary Information

Supplementary Movie 1

Supplementary Movie 2

Peer Review File

## References

[b1] Goldblum J. R., Rice T. W. & Richter J. E. Histopathologic features in esophagomyotomy specimens from patients with achalasia. Gastroenterology 111, 648–654 (1996). 8780569 10.1053/gast.1996.v111.pm8780569

[b2] Knowles C. H. . New perspectives in the diagnosis and management of enteric neuropathies. Nat. Rev. Gastroenterol. Hepatol. 10, 206–218 (2013). 23399525 10.1038/nrgastro.2013.18

[b3] Csendes A. . Histological studies of Auerbach’s plexuses of the oesophagus, stomach, jejunum, and colon in patients with achalasia of the oesophagus: correlation with gastric acid secretion, presence of parietal cells and gastric emptying of solids. Gut 33, 150–154 (1992). 1541407 10.1136/gut.33.2.150 PMC1373920

[b4] Hasler W. L. Gastroparesis: pathogenesis, diagnosis and management. Nat. Rev. Gastroenterol. Hepatol. 8, 438–453 (2011). 21769117 10.1038/nrgastro.2011.116

[b5] Harberson J. . Gastric neuromuscular pathology in gastroparesis: analysis of full-thickness antral biopsies. Dig. Dis. Sci. 55, 359–370 (2010). 19997975 10.1007/s10620-009-1071-2

[b6] Giorgio V. . High-resolution colonic manometry accurately predicts colonic neuromuscular pathological phenotype in pediatric slow transit constipation. Neurogastroenterol. Motil. 25, 70–8.e8–9 (2013). 23030503 10.1111/nmo.12016

[b7] Rao S. S., Rattanakovit K. & Patcharatrakul T. Diagnosis and management of chronic constipation in adults. Nat. Rev. Gastroenterol. Hepatol. 13, 295–305 (2016). 27033126 10.1038/nrgastro.2016.53

[b8] Bassotti G. . Enteric neuropathology of the terminal ileum in patients with intractable slow-transit constipation. Hum. Pathol. 37, 1252–1258 (2006). 16949932 10.1016/j.humpath.2006.04.027

[b9] Heanue T. A. & Pachnis V. Enteric nervous system development and Hirschsprung’s disease: advances in genetic and stem cell studies. Nat. Rev. Neurosci. 8, 466–479 (2007). 17514199 10.1038/nrn2137

[b10] Amiel J. . Hirschsprung disease, associated syndromes and genetics: a review. J. Med. Genet. 45, 1–14 (2008). 17965226 10.1136/jmg.2007.053959

[b11] Rao M. & Gershon M. D. The bowel and beyond: the enteric nervous system in neurological disorders. Nat. Rev. Gastroenterol. Hepatol. 13, 517–528 (2016). 27435372 10.1038/nrgastro.2016.107 PMC5005185

[b12] Anderson G. . Loss of enteric dopaminergic neurons and associated changes in colon motility in an MPTP mouse model of Parkinson’s disease. Exp. Neurol. 207, 4–12 (2007). 17586496 10.1016/j.expneurol.2007.05.010 PMC2277100

[b13] Joachim C. L., Mori H. & Selkoe D. J. Amyloid beta-protein deposition in tissues other than brain in Alzheimer’s disease. Nature 341, 226–230 (1989). 2528696 10.1038/341226a0

[b14] Burns A. J. & Thapar N. Neural stem cell therapies for enteric nervous system disorders. Nat. Rev. Gastroenterol. Hepatol. 11, 317–328 (2014). 24322895 10.1038/nrgastro.2013.226

[b15] Bondurand N. . Neuron and glia generating progenitors of the mammalian enteric nervous system isolated from foetal and postnatal gut cultures. Development 130, 6387–6400 (2003). 14623827 10.1242/dev.00857

[b16] Cooper J. E. . *In vivo* transplantation of enteric neural crest cells into mouse gut; engraftment, functional integration and long-term safety. PLoS ONE 11, e0147989 (2016). 26824433 10.1371/journal.pone.0147989 PMC4732685

[b17] Hotta R. . Transplanted progenitors generate functional enteric neurons in the postnatal colon. J. Clin. Invest. 123, 1182–1191 (2013). 23454768 10.1172/JCI65963 PMC3582137

[b18] Cooper J. E. . *In vivo* transplantation of fetal human gut-derived enteric neural crest cells. Neurogastroenterol. Motil. 29, e12900 (2017). 10.1111/nmo.12900 PMC5215633 27380932

[b19] Hetz S. . *In vivo* transplantation of neurosphere-like bodies derived from the human postnatal and adult enteric nervous system: a pilot study. PLoS ONE 9, e93605 (2014). 24699866 10.1371/journal.pone.0093605 PMC3974735

[b20] Nishikawa R. . Migration and differentiation of transplanted enteric neural crest-derived cells in murine model of Hirschsprung’s disease. Cytotechnology 67, 661–670 (2015). 25230796 10.1007/s10616-014-9754-8 PMC4474987

[b21] Metzger M. . Enteric nervous system stem cells derived from human gut mucosa for the treatment of aganglionic gut disorders. Gastroenterology 136, 2214–25 e1-3 (2009). 19505425 10.1053/j.gastro.2009.02.048

[b22] Rivera L. R. . The involvement of nitric oxide synthase neurons in enteric neuropathies. Neurogastroenterol. Motil. 23, 980–988 (2011). 21895878 10.1111/j.1365-2982.2011.01780.x

[b23] De Giorgio R. . Esophageal and gastric nitric oxide synthesizing innervation in primary achalasia. Am. J. Gastroenterol. 94, 2357–2362 (1999). 10483991 10.1111/j.1572-0241.1999.01357.x

[b24] Vanderwinden J. M. . Nitric oxide synthase activity in infantile hypertrophic pyloric stenosis. N. Engl J. Med. 327, 511–515 (1992). 1378938 10.1056/NEJM199208203270802

[b25] Grover M. . Cellular changes in diabetic and idiopathic gastroparesis. Gastroenterology 140, 1575–85 e8 (2011). 21300066 10.1053/j.gastro.2011.01.046 PMC3081914

[b26] Chandrasekharan B. & Srinivasan S. Diabetes and the enteric nervous system. Neurogastroenterol. Motil. 19, 951–960 (2007). 17971027 10.1111/j.1365-2982.2007.01023.x PMC3711013

[b27] Kusafuka T. & Puri P. Altered mRNA expression of the neuronal nitric oxide synthase gene in Hirschsprung’s disease. J. Pediatr. Surg. 32, 1054–1058 (1997). 9247233 10.1016/s0022-3468(97)90398-5

[b28] Vanderwinden J. M. . Nitric oxide synthase distribution in the enteric nervous system of Hirschsprung’s disease. Gastroenterology 105, 969–973 (1993). 7691675 10.1016/0016-5085(93)90938-9

[b29] Mashimo H., Kjellin A. & Goyal R. K. Gastric stasis in neuronal nitric oxide synthase-deficient knockout mice. Gastroenterology 119, 766–773 (2000). 10982771 10.1053/gast.2000.16509

[b30] Micci M. A. . Neural stem cell transplantation in the stomach rescues gastric function in neuronal nitric oxide synthase-deficient mice. Gastroenterology 129, 1817–1824 (2005). 16344050 10.1053/j.gastro.2005.08.055

[b31] Huang P. L. . Targeted disruption of the neuronal nitric oxide synthase gene. Cell 75, 1273–1286 (1993). 7505721 10.1016/0092-8674(93)90615-w

[b32] Dickson E. J. . The mechanisms underlying the generation of the colonic migrating motor complex in both wild-type and nNOS knockout mice. Am. J. Physiol. Gastrointest. Liver Physiol. 298, G222–G232 (2010). 19959818 10.1152/ajpgi.00399.2009 PMC2822500

[b33] Ward S. M. . Nonadrenergic, noncholinergic inhibition and rebound excitation in canine colon depend on nitric oxide. Am. J. Physiol. 262, G237–G243 (1992). 1539659 10.1152/ajpgi.1992.262.2.G237

[b34] Franck H. . Rebound excitation and alternating slow wave patterns depend upon eicosanoid production in canine proximal colon. J. Physiol. 520, (Pt 3): 885–895 (1999). 10545151 10.1111/j.1469-7793.1999.00885.x PMC2269616

[b35] Sanders K. M. . Development and plasticity of interstitial cells of Cajal. Neurogastroenterol. Motil. 11, 311–338 (1999). 10520164 10.1046/j.1365-2982.1999.00164.x

[b36] Ward S. M. . Mutation of the proto-oncogene c-kit blocks development of interstitial cells and electrical rhythmicity in murine intestine. J. Physiol. 480, (Pt 1): 91–97 (1994). 7853230 10.1113/jphysiol.1994.sp020343 PMC1155780

[b37] Huizinga J. D. . W/kit gene required for interstitial cells of Cajal and for intestinal pacemaker activity. Nature 373, 347–349 (1995). 7530333 10.1038/373347a0

[b38] Hennig G. W. . ICC-MY coordinate smooth muscle electrical and mechanical activity in the murine small intestine. Neurogastroenterol. Motil. 22, e138–e151 (2010). 20059699 10.1111/j.1365-2982.2009.01448.x PMC2856807

[b39] Burns A. J. . Interstitial cells of Cajal mediate inhibitory neurotransmission in the stomach. Proc. Natl Acad. Sci. USA 93, 12008–12013 (1996). 8876253 10.1073/pnas.93.21.12008 PMC38174

[b40] Ward S. M. . Interstitial cells of Cajal mediate cholinergic neurotransmission from enteric motor neurons. J. Neurosci. 20, 1393–1403 (2000). 10662830 10.1523/JNEUROSCI.20-04-01393.2000 PMC6772355

[b41] Klein S. . Interstitial cells of Cajal integrate excitatory and inhibitory neurotransmission with intestinal slow-wave activity. Nat. Commun. 4, 1630 (2013). 23535651 10.1038/ncomms2626

[b42] Iino S., Horiguchi K. & Nojyo Y. Interstitial cells of Cajal are innervated by nitrergic nerves and express nitric oxide-sensitive guanylate cyclase in the guinea-pig gastrointestinal tract. Neuroscience 152, 437–448 (2008). 18280665 10.1016/j.neuroscience.2007.12.044

[b43] Lies B. . Nitrergic signalling via interstitial cells of Cajal regulates motor activity in murine colon. J. Physiol. 593, 4589–4601 (2015). 26227063 10.1113/JP270511 PMC4606533

[b44] Choi K. M. . Regulation of interstitial cells of Cajal in the mouse gastric body by neuronal nitric oxide. Neurogastroenterol. Motil. 19, 585–595 (2007). 17593140 10.1111/j.1365-2982.2007.00936.x

[b45] Binder E. . Enteric neurospheres are not specific to neural crest cultures: implications for neural stem cell therapies. PLoS ONE 10, e0119467 (2015). 25799576 10.1371/journal.pone.0119467 PMC4370605

[b46] Torihashi S. . Enteric neurons express steel factor-lacZ transgene in the murine gastrointestinal tract. Brain Res. 738, 323–328 (1996). 8955529 10.1016/s0006-8993(96)00935-3

[b47] Young H. M. . Identification of neurons that express stem cell factor in the mouse small intestine. Gastroenterology 115, 898–908 (1998). 9753493 10.1016/s0016-5085(98)70262-8

[b48] Fattahi F. . Deriving human ENS lineages for cell therapy and drug discovery in Hirschsprung disease. Nature 531, 105–109 (2016). 26863197 10.1038/nature16951 PMC4846424

[b49] Schindelin J. . Fiji: an open-source platform for biological-image analysis. Nat. Methods 9, 676–682 (2012). 22743772 10.1038/nmeth.2019 PMC3855844

