# Supplementary material for: Transplantation of enteric nervous system stem cells rescues nitric oxide synthase deficient mouse colon
Source: Nat Commun. 2017 Jul 3;8:15937. doi: 10.1038/ncomms15937 (PMC5500880; doi:10.1038/ncomms15937)
Supplement: Supplementary Information [file ncomms15937-s1.pdf]

Type of file: pdf
Size of file: 0 KB
Title of file for HTML: Supplementary Information
Description: Supplementary Figures and Supplementary Tables

Type of file: pdf
Size of file: 0 KB
Title of file for HTML: Peer Review File
Description:

Type of file: mp4
Size of file: 0 KB
Title of file for HTML: Supplementary Movie 1
Description: Transplanted ENSC integrate and form extensive networks in the nNOS-/- Colon. Video of montaged image (Supplementary Fig. 2) demonstrating integration of transplanted ENSC in the nNOS-/- Colon. Transplanted cells form extensive anastamosing networks extending in both oral and anal directions from the transplant site.

Type of file: mp4
Size of file: 0 KB
Title of file for HTML: Supplementary Movie 2
Description: Transplanted ENSC integrate within ganglia along the length of the nNOS-/- Colon. Video of stitched confocal z-stack image (Supplementary Fig. 3) demonstrating integration of transplanted ENSC within endogenous ganglia along the length of the nNOS-/- Colon.

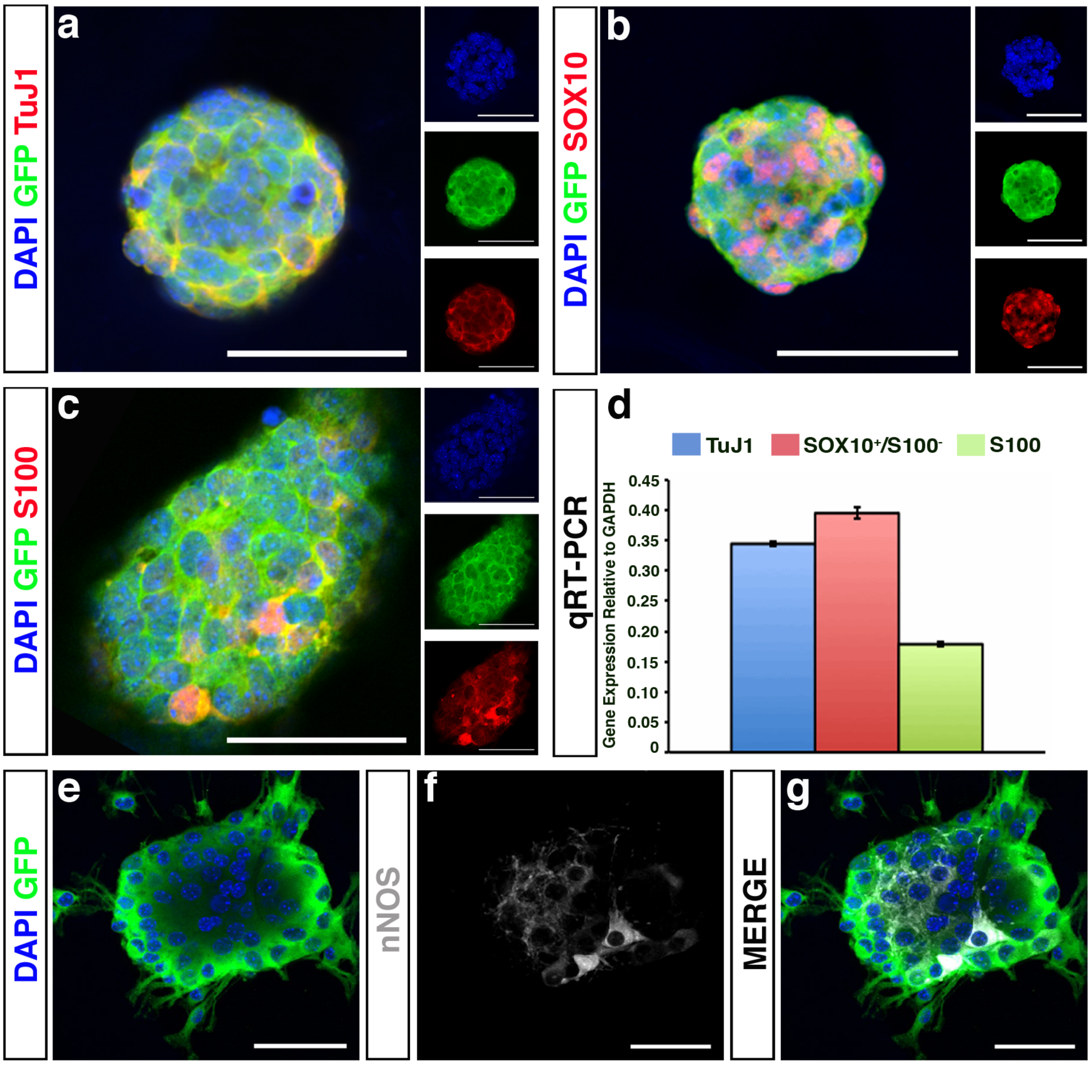


**Supplementary Fig. 1.** *Wnt1*$^{cre/+}$*;R26R*$^{YFP/YFP}$ derived neurospheres contain critical ENS cells. (**a-c**) Representative confocal images of *Wnt1*$^{cre/+}$*;R26R*$^{YFP/YFP}$ derived neurospheres. *Wnt1*$^{cre/+}$*;R26R*$^{YFP/YFP}$ derived GFP$^{+}$ neurospheres co-label with ENS markers for neurons (TuJ1; a), ENSC (SOX10; b) and glia (S100; c). Small images are of individual channels taken form (a-c). Dapi was used to label nuclei. (**d**) Quantitative gene expression of neurons (TuJ1$^{+}$), ENSC (SOX10$^{+}$S100$^{-}$) and glia (S100$^{+}$) relative to GAPDH within a neurosphere population. n=6 neurospheres. Error bars represent mean ± s.e.m. in all panels.

(**e-g**) Representative confocal images of a GFP$^{+}$ neurosphere (e) co-labeled with nNOS (f). Dapi was used to label nuclei. Merged image (g) demonstrates the ability

of *Wnt1*$^{cre/+}$;*R26R*$^{YFP/YFP}$ derived cells to form nNOS$^{+}$ neurons, *in vitro*, prior to transplantation. Scale bars, 50μm.

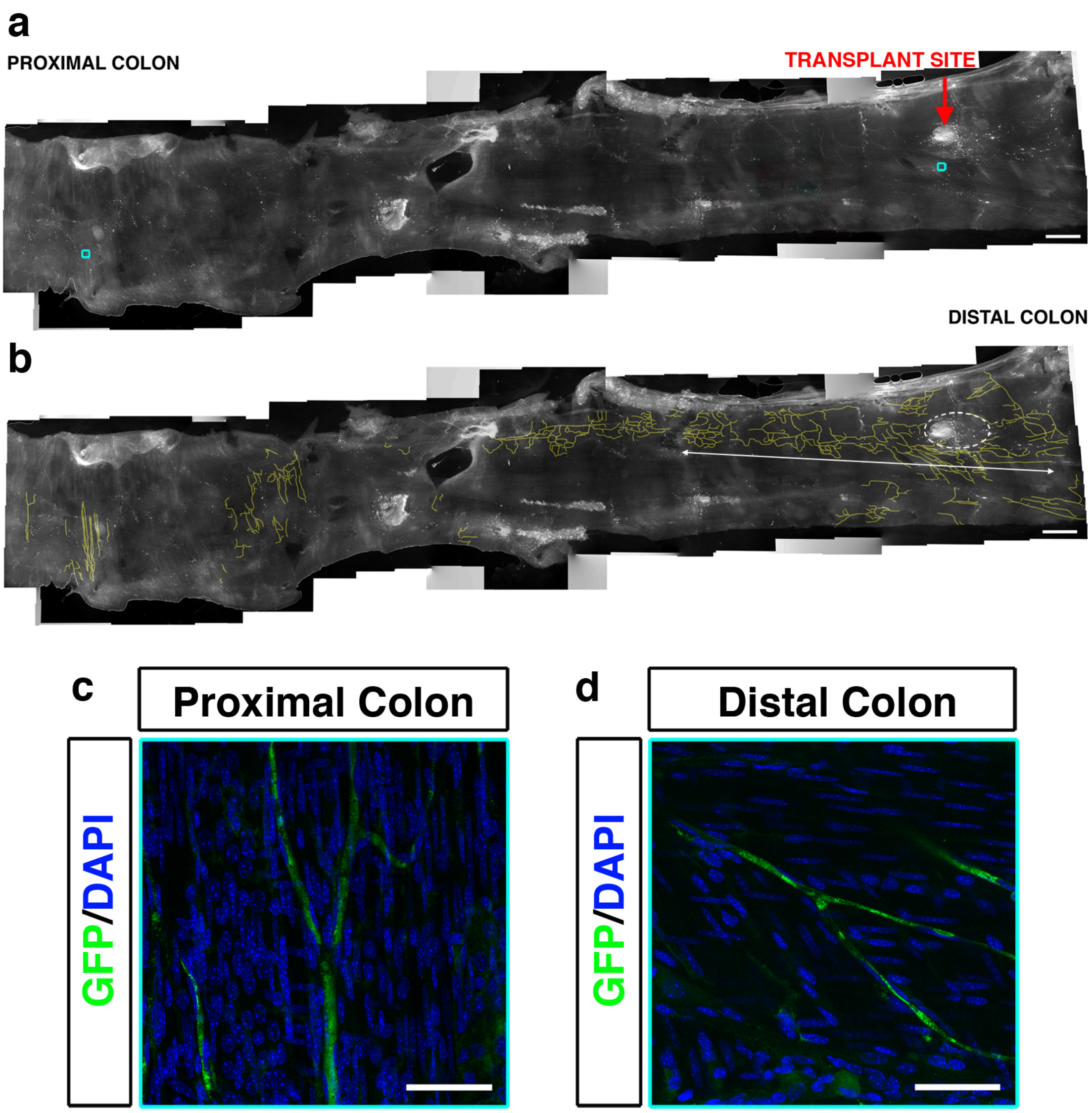


**Supplementary Fig. 2.** ENSC derived cells integrate along the length of the colon. (**a**) Montage of 89 fluorescent images of *nNOS*$^{-/-}$ colon 4 weeks after transplantation. GFP$^{+}$ cells and fibres integrate into both the distal and proximal colon, see Supplementary Video 1, at significant distances from the transplant site. Scale bar, 1000µm. (**b**) Image from (a) with superimposed map of transplanted cells in yellow. The presumptive transplant site is delineated with a dashed line. A dense network of GFP$^{+}$ cells can be observed at this site with donor cells forming anastomosing networks in both oral and aboral directions. The maximum distance recorded for continuously networked cells was 10.79mm (white arrow). (**c-d**) High power confocal

images of highlighted regions in (a) demonstrating $GFP^+$ transplanted cells within the proximal (c) and distal (d) colon. Scale bars, 50µm (**c,d**).

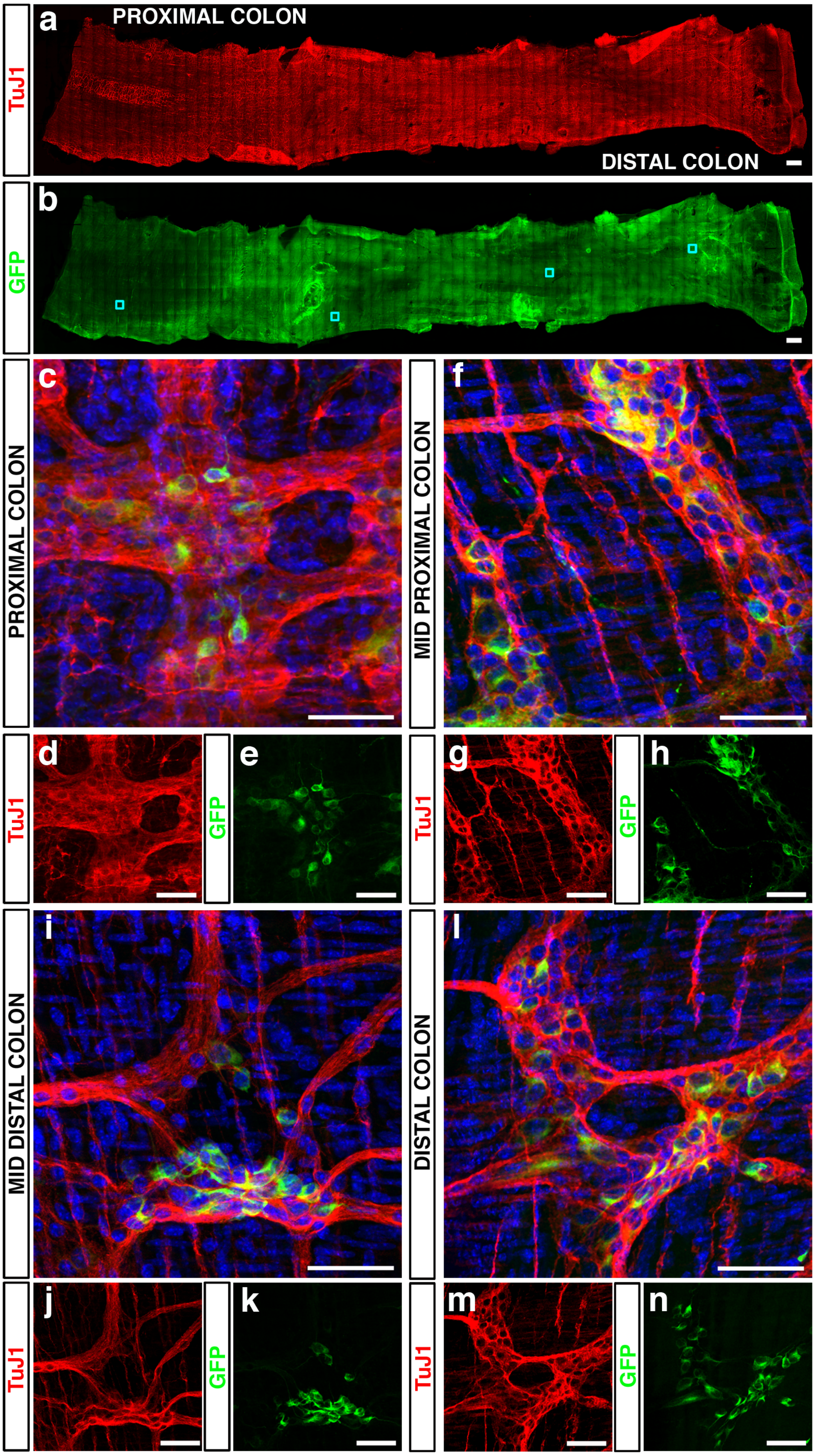
a
PROXIMAL COLON
DISTAL COLON
TuJ1
b
GFP
c
PROXIMAL COLON
f
MID PROXIMAL COLON
d
TuJ1
e
GFP
g
TuJ1
h
GFP
i
MID DISTAL COLON
l
DISTAL COLON
j
TuJ1
k
GFP
m
TuJ1
n
GFP

**Supplementary Fig. 3.** ENSC derived cells integrate within enteric ganglia along the length of the colon. (**a,b**) Overview of stitched confocal z-stack images (16884 total images) taken from transplanted *nNOS*$^{-/-}$ colon 4 weeks after transplantation labeled with TuJ1 (a) and GFP (b). GFP$^{+}$ cells integrate along the entire length of the colon, see Supplementary Video 2. Scale bar, 1000μm. (**c-n**) Representative high power confocal images of highlighted inset regions from (a) demonstrating GFP$^{+}$ transplanted cells within the proximal (c-e), mid proximal (f-h), mid distal (i-k) and distal (l-n) colon. Transplanted cells (green) integrate within endogenous ganglia and co-express Tuj1 (red). Scale bars, 50μm (**c-n**).

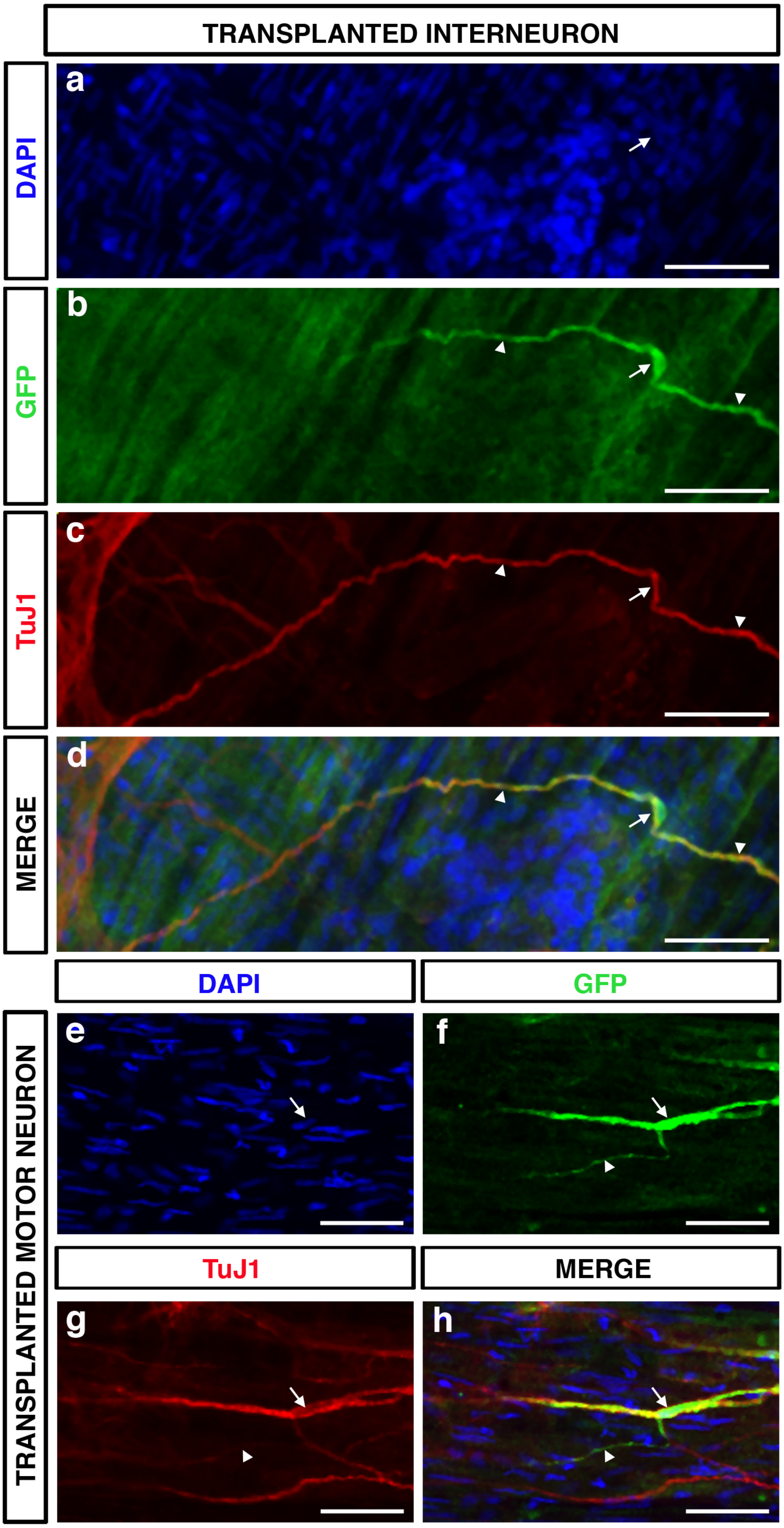
TRANSPLANTED INTERNEURON
DAPI
GFP
TuJ1
MERGE
a
b
c
d
DAPI
GFP
TRANSPLANTED MOTOR NEURON
e
f
TuJ1
MERGE
g
h

**Supplementary Fig. 4.** ENSC derived cells form interneurons and motor neurons in the transplanted *nNOS*$^{-/-}$ colon. (**a-d**) Representative confocal z-stack images demonstrating a transplanted cell with morphological characteristics of an interneuron. Individual channels for DAPI (a), GFP (b), and TuJ1 (c) within a transplanted cell present in the *nNOS*$^{-/-}$ distal colon. (**d)** Merged image of individual channels shown in (a-c). A transplanted cell (arrow) could be observed extending bipolar processes (arrowheads) characteristic of an enteric interneuron making connection with the endogenous neural network at the level on the myenteric plexus. (**e-h**) Confocal z-stack images demonstrating a transplanted cell with morphological characteristics of an enteric motor neuron. Individual channels for DAPI (e), GFP (f) and TuJ1 (g) within a transplanted cell present in the *nNOS*$^{-/-}$ colon. (**h)** Merged image of individual channels shown in (e-g). Transplanted neuron (arrow), co-expressing GFP (green) and TuJ1 (red) were observed extending multiple processes. These processes appear to align with colonic muscle fibres (arrowheads) characteristic of an enteric motor neuron. Scale bars, 20μm

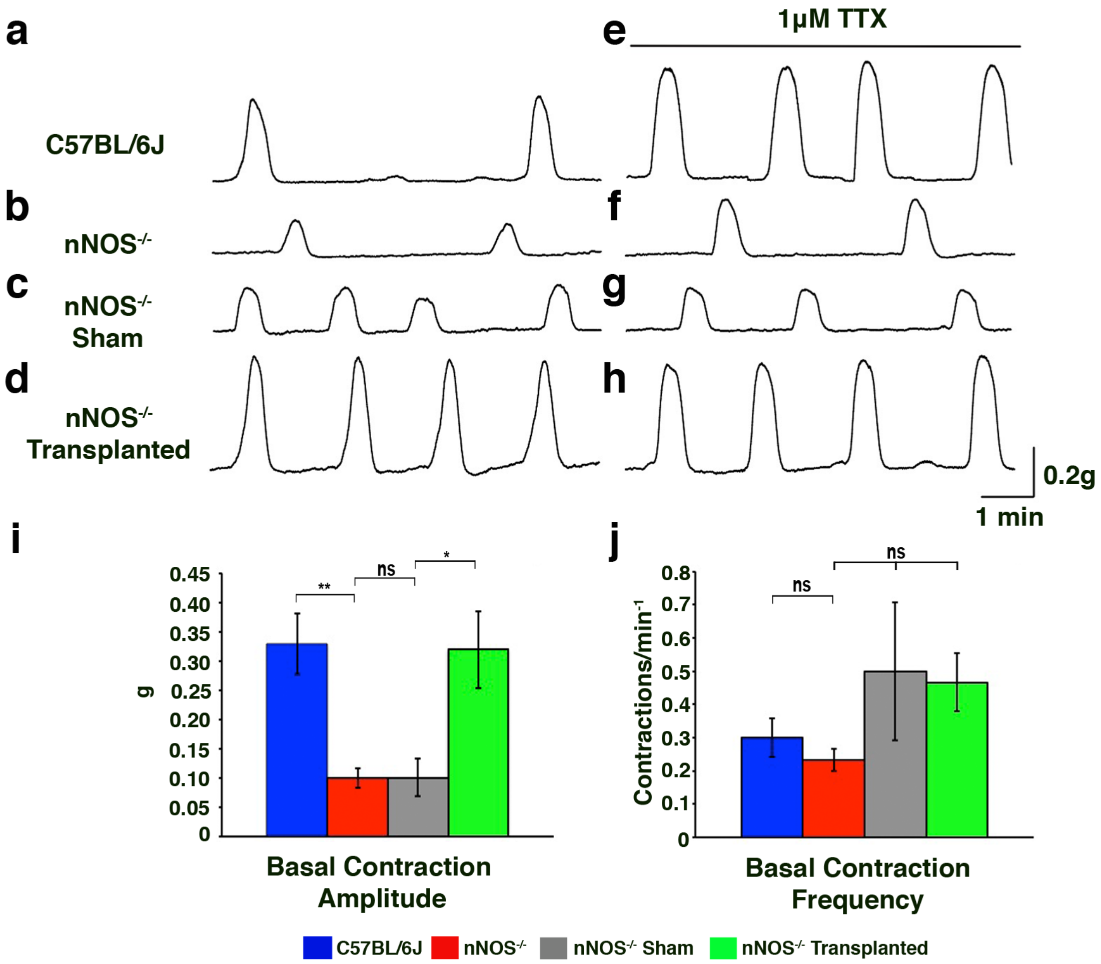


**Supplementary Fig. 5.** ENSC transplantation to the distal colon alters the contractile properties of the proximal colon**.** (**a-d**) Representative organ bath contractility traces demonstrating the basal contractile patterns of C57BL/6J, *nNOS*$^{-/-}$, sham-operated *nNOS*$^{-/-}$, and transplanted *nNOS*$^{-/-}$ proximal colon in control conditions. (**e-h**) Representative organ bath contractility traces showing basal contractile patterns of C57BL/6J, *nNOS*$^{-/-}$, sham-operated *nNOS*$^{-/-}$, and transplanted *nNOS*$^{-/-}$ distal colon from (a-d) after the addition of TTX. (**i**) Quantification of basal contractile amplitude in C57BL/6J (blue bars), *nNOS*$^{-/-}$ (red bars), sham-operated *nNOS*$^{-/-}$ (grey bars) and transplanted *nNOS*$^{-/-}$ (green bars) distal colon. n=5 for each group. $^{**}P \leq 0.01$, $^{*}P \leq 0.05$ by Student's t-test. (**j**) Quantification of basal contractile frequency in C57BL/6J (blue bars), *nNOS*$^{-/-}$ (red bars), sham-operated *nNOS*$^{-/-}$ (grey bars) and transplanted

*nNOS*$^{-/-}$ (green bars) distal colon. n=5 for each group. Error bars represent mean ± s.e.m. in all panels.

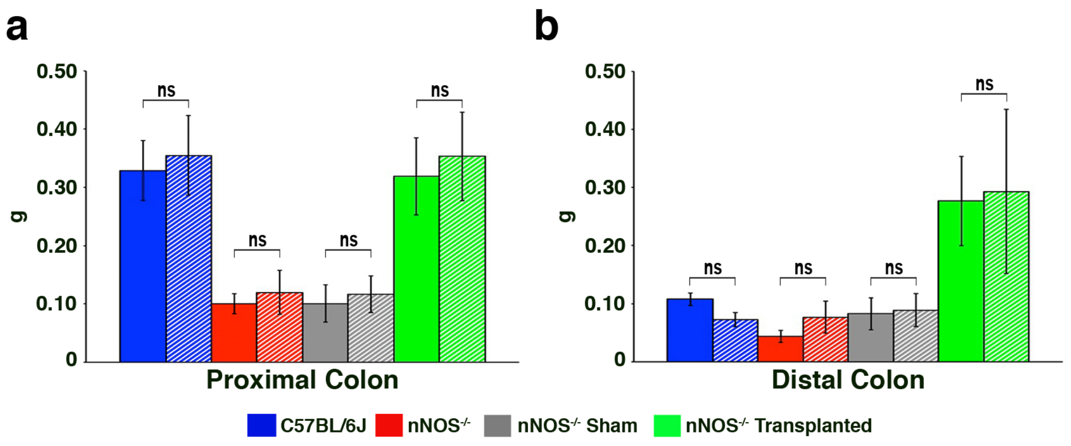


**Supplementary Fig. 6.** Increased basal contractile patterns in transplanted *nNOS*$^{-/-}$ mice are not neurally mediated. **(a,b)** Quantification of the effect of TTX administration on basal contractile amplitude in the proximal (a) and distal colon (b) of C57BL/6J, *nNOS*$^{-/-}$, sham-operated *nNOS*$^{-/-}$, and transplanted *nNOS*$^{-/-}$ mice. **(a)** TTX (striped bars) had no effect on contractile amplitude (solid bars) in the proximal colon of C57BL/6J (0.328±0.051g vs 0.354±0.068g, n=3, P=0.775, blue bars), *nNOS*$^{-/-}$ (0.1±0.017g vs 0.12±0.038g, n=3, P=0.656, red bars), sham-operated *nNOS*$^{-/-}$ (0.1±0.032g vs 0.12±0.031g, n=3, P=0.677, grey bars) and transplanted *nNOS*$^{-/-}$ (0.319±0.066g vs 0.353±0.076g, n=3, P=0.753, green bars) mice. **(b)** TTX similarly had no effect on contractile amplitude in distal colon segments of C57BL/6J (0.108±0.011g; vs 0.072±0.01g; n=3, P=0.0921, blue bars), *nNOS*$^{-/-}$ (0.044±0.011g; vs 0.077±0.027g; n=3, P=0.322, red bars), sham-operated *nNOS*$^{-/-}$ (0.083±0.028g vs 0.089±0.029g; n=3, P=0.891, grey bars) and transplanted *nNOS*$^{-/-}$ (0.277±0.077g vs 0.293±0.14g; n=3, P=0.925, green bars) mice. Error bars represent mean ± s.e.m. in all panels.

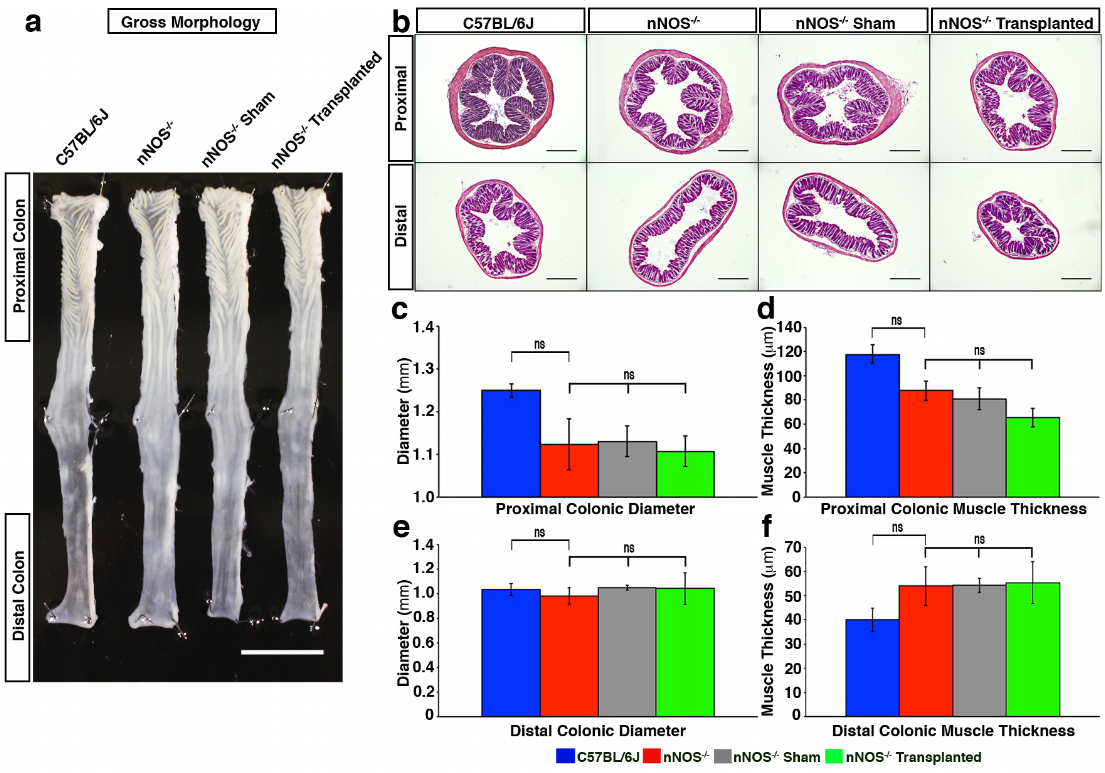


**Supplementary Fig. 7.** Colonic morphology is unaffected by ENSC transplantation. **(a)** Representative images demonstrating gross morphology of C57BL/6J, *nNOS*$^{-/-}$, sham-operated *nNOS*$^{-/-}$, and transplanted *nNOS*$^{-/-}$ colon tissues. No evidence of an inflammatory response was observed within transplanted *nNOS*$^{-/-}$ colon. Scale bars, 10mm. (**b**) Representative H&E sections of both proximal and distal colon from C57BL/6J, *nNOS*$^{-/-}$, sham-operated *nNOS*$^{-/-}$, and transplanted *nNOS*$^{-/-}$ mice. Scale bars, 500µm. (**c-f)** Quantification of colonic diameter and muscle thickness. (**c,d**) No differences were observed between the diameter shown in (c) of the proximal colon between C57BL/6J (117.67±7.67µm, n=3, blue bars) and *nNOS*$^{-/-}$ mice (87.67±7.88µm; n=3, red bars; P>0.05), or muscle thickness shown in (d) in C57BL/6J (1.25±0.02mm; n=3) compared to *nNOS*$^{-/-}$ mice (1.12±0.06mm; n=3; P>0.05). Additionally, the proximal colon of non-transplanted *nNOS*$^{-/-}$, sham operated *nNOS*$^{-/-}$ (grey bars) and transplanted *nNOS*$^{-/-}$ (green bars) animals were similar in terms of diameter (F(2,6)=0.07; P>0.05) and muscle thickness (F(2,6)=1.95, P>0.05)

as determined by one-way ANOVA. (**e**) Equivalent histological analysis of distal colonic regions similarly revealed no significant changes in the diameter, of C57BL/6J distal colon (1.03±0.08mm; n=3) to that of *nNOS*$^{-/-}$ mice (1.12±0.68mm; n=3; P>0.05). Similar to the proximal colon, no differences were observed in the diameter of the distal colon between transplanted *nNOS*$^{-/-}$ mice (1.04±0.13mm; n=3) compared to either non-transplanted *nNOS*$^{-/-}$ (1.12±0.68mm; n=3) or sham-operated *nNOS*$^{-/-}$ (1.05±0.02mm; n=3) as determined by one way ANOVA (F(2,6)=0.19, P>0.05). (**f**) No change was observed between the muscle thickness of C57BL/6J distal colon (48.00±4.93µm; n=3) and that of *nNOS*$^{-/-}$ mice (54.00±8.00µm; n=3; P>0.05). Similarly, no change was observed in distal muscle thickness between the group means (F(2,6)=0.01, P>0.05) of distal muscle thickness in transplanted *nNOS*$^{-/-}$ mice (55.33±8.67µm; n=3) compared to either non-transplanted *nNOS*$^{-/-}$ (54.0±8.0µm; n=3) or sham-operated *nNOS*$^{-/-}$ (54.33±2.96µm; n=3). Error bars represent mean ± s.e.m. in all panels.

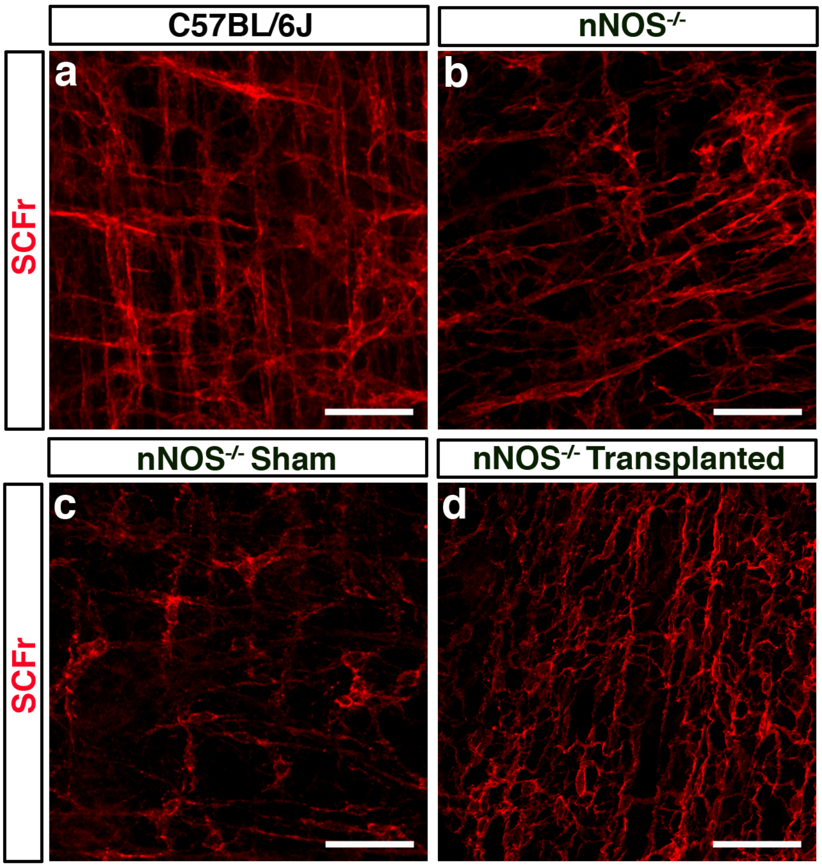


**Supplementary Fig. 8.** ENSC transplantation alters ICC networks in *nNOS*$^{-/-}$ proximal colon. (**a-d**) Representative z-stack confocal images of ICC in C57BL/6J, *nNOS*$^{-/-}$, sham-operated *nNOS*$^{-/-}$, and transplanted *nNOS*$^{-/-}$ proximal colon. Scale bars, 50µm. Summary data of ICC numbers within proximal colonic segments are included in Fig. 6f.

**Supplementary Table 1. Primer Sequences used for RT-PCR**

| Gene | Accession number | Primer Sequences |
|---|---|---|
| GAPDH | NM_008084.3 | for: TCCTGCACCACCAACTGCTT<br>rev: CACGCCACAGCTTTCCAGAG |
| TuJ-1 | NM_023279.2 | for: CAGGGCCATTCTGGTGGACT<br>rev: TAGTGCCCTTTGGCCCAGTT |
| Sox10 | NM_011437 | for: AGCCGACCAGTACCCTCACC<br>rev: GGAGCCTCTCAGCCTCCTCA |
| S100β | NM_009115 | for: GCGAGAGGGTGACAAGCACA<br>rev: CGTCCAGCGTCTCCATCACT |
| nNOS | NM_008712 | for: ATCTGTCTCGCCAGCCATCAGCCA<br>rev: GGAGCTTTGTGCAGTTTGCCGTCG |
| SCF Ligand | NM_013598 | for: GAAGTCAGTCTTTTCCCTTGACAGT<br>rev:GCATGTCACATTATACTATTGCAAACA |

**Supplementary Table 2. Primary Antibodies used for Immunohistochemistry**

| PRIMARY ANTIBODY | CONCENTRATION | COMPANY |
|---|---|---|
| Mouse anti-TuJ1 | 1:500 | Covance |
| Rabbit anti-GFP | 1:500 | Invitrogen |
| Goat anti-Sox10 | 1:300 | Santa Cruz |
| Rabbit anti-S100 | 1:400 | Dako |
| Rabbit anti-nNOS | 1:400 | Invitrogen |
| Goat anti-SCFr | 1:500 | R&D Systems |
| Rat anti-BRDU | 1:20 | Oxford Biotech |

**Supplementary Table 3. Secondary Antibodies used for Immunohistochemistry**

| SECONDARY ANTIBODY | ALEXA FLUOR | CONCENTRATION | COMPANY |
|---|---|---|---|
| Goat anti-mouse | 488 | 1:500 | Invitrogen |
| Goat anti-mouse | 568 | 1:500 | Invitrogen |
| Goat anti-rabbit | 568 | 1:500 | Invitrogen |
| Goat anti-rabbit | 647 | 1:500 | Invitrogen |
| Donkey anti-goat | 568 | 1:500 | Invitrogen |
| Goat anti-Rat | 647 | 1:500 | Invitrogen |
| DAPI | | 1:1000 | Sigma |
